# Supplementary material for: Genetic ancestry and ethnic identity in Ecuador
Source: HGG Adv. 2021 Aug 20;2(4):100050. doi: 10.1016/j.xhgg.2021.100050 (PMC8756502; doi:10.1016/j.xhgg.2021.100050)
Supplement: Document S1. Figures S1–S9 and Table S1 [file mmc1.pdf]

HGGA, Volume *2*

Supplemental information

Genetic ancestry and ethnic identity in Ecuador

Shashwat Deepali Nagar, Andrew B. Conley, Aroon T. Chande, Lavanya Rishishwar, Shivam Sharma, Leonardo Mariño-Ramírez, Gabriela Aguinaga-Romero, Fabricio González-Andrade, and I. King Jordan

Supplementary material

Genetic ancestry and ethnic identity in Ecuador

Shashwat D. Nagar, Andrew B. Conley, Aroon T. Chande, Lavanya Rishishwar, Shivam Sharma, Leonardo Mariño-Ramírez, Gabriela Aguinaga-Romero, Fabricio González-Andrade, and I. King Jordan

Figure S1. Genotype data harmonization and analysis workflow. 2

Figure S2. Principal component analysis (PCA) of the genomic relationship matrix for the four Ecuadorian populations compared to reference populations from Africa, the Americas, East Asia, and Europe. 3

Figure S3. ADMIXTURE plots showing continental ancestry fractions for individuals from (A) African, Admixed American, East Asian, and European reference populations and (B) the four Ecuadorian ethnic groups. 4

Figure S4. Sex-biased admixture for Tsáchila subgroups. 5

Figure S5. African reference populations used for this study. 6

Figure S6. Validation of African subcontinental ancestry inference. 7

Figure S7. Native American reference populations used for this study. 8

Figure S8. Cross-validation error values (y-axis) for AMDIXTURE run over a range of ancestry component values K=2-12 on Native American ancestry.. 9

Figure S9. Native American origins of Ecuadorian populations. 10

Table S1. Reference populations used in this study. 11

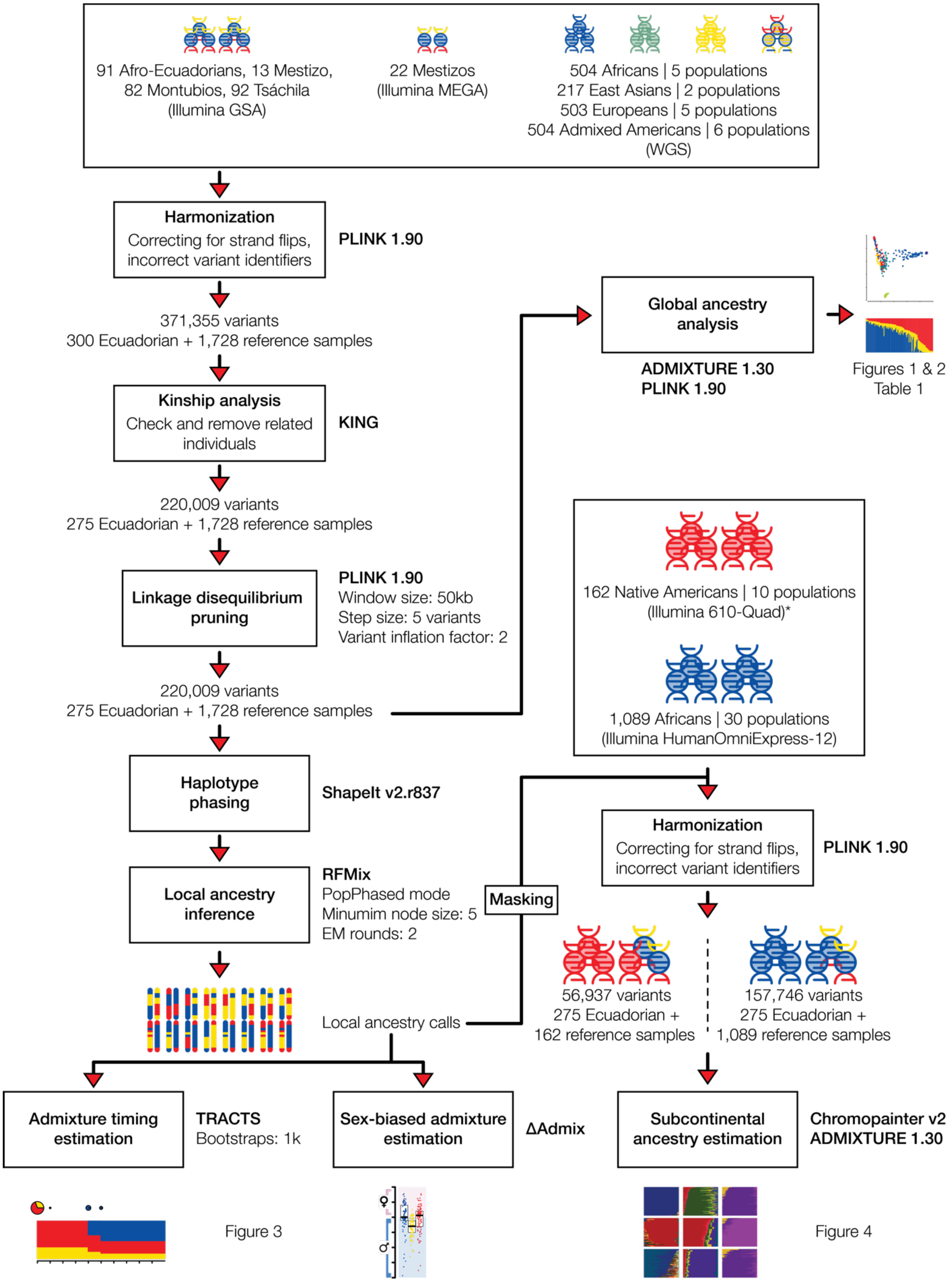

Figure S1. **Genotype data harmonization and analysis workflow.** Different datasets, harmonization and analysis steps, along with tools used at each step are illustrated in this workflow.

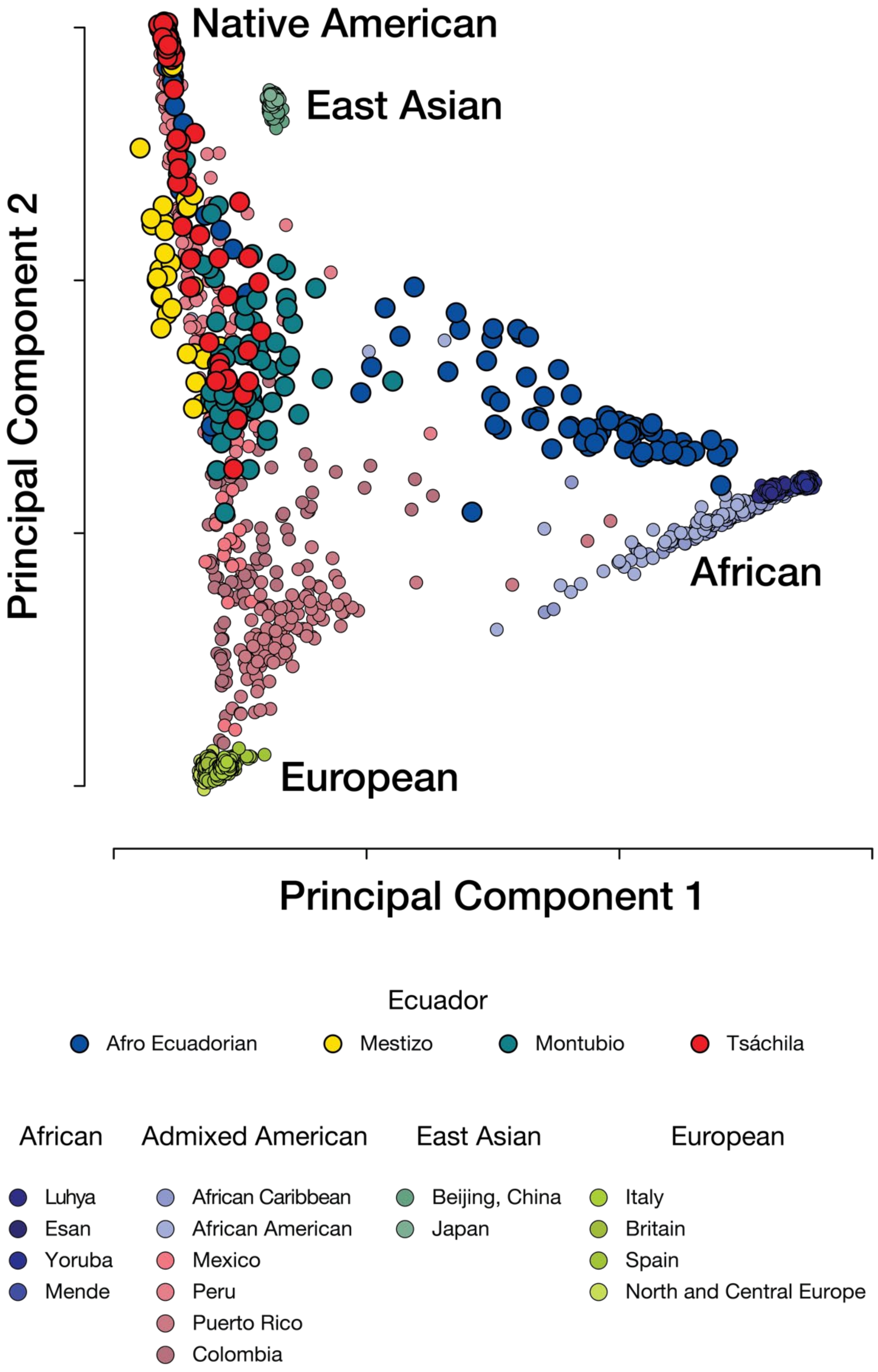

Figure S2. **Principal component analysis (PCA) of the genomic relationship matrix for the four Ecuadorian populations compared to reference populations from Africa, the Americas, East Asia, and Europe.**

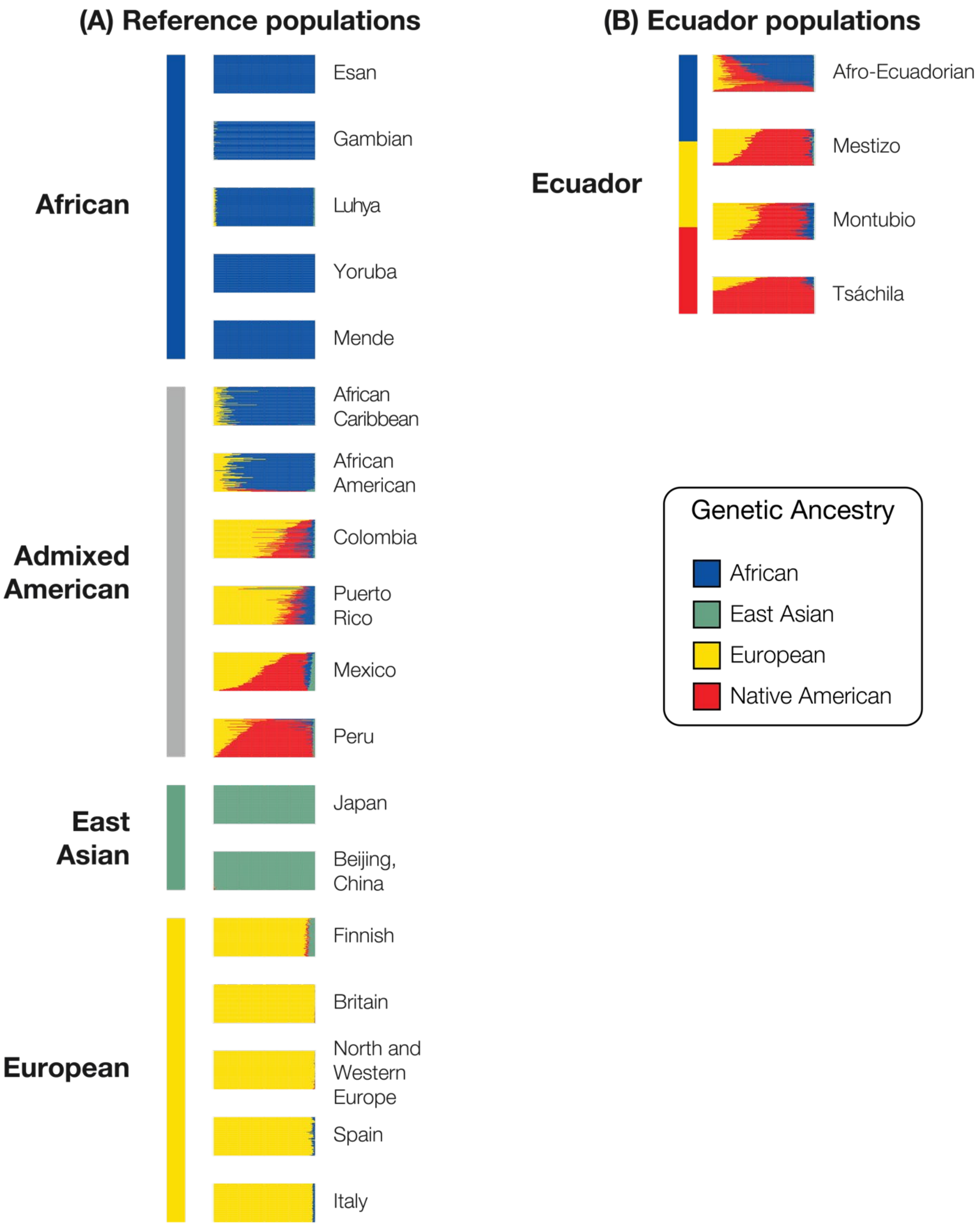

Figure S3. **ADMIXTURE plots showing continental ancestry fractions for individuals from (A) African, Admixed American, East Asian, and European reference populations and (B) the four Ecuadorian ethnic groups.**

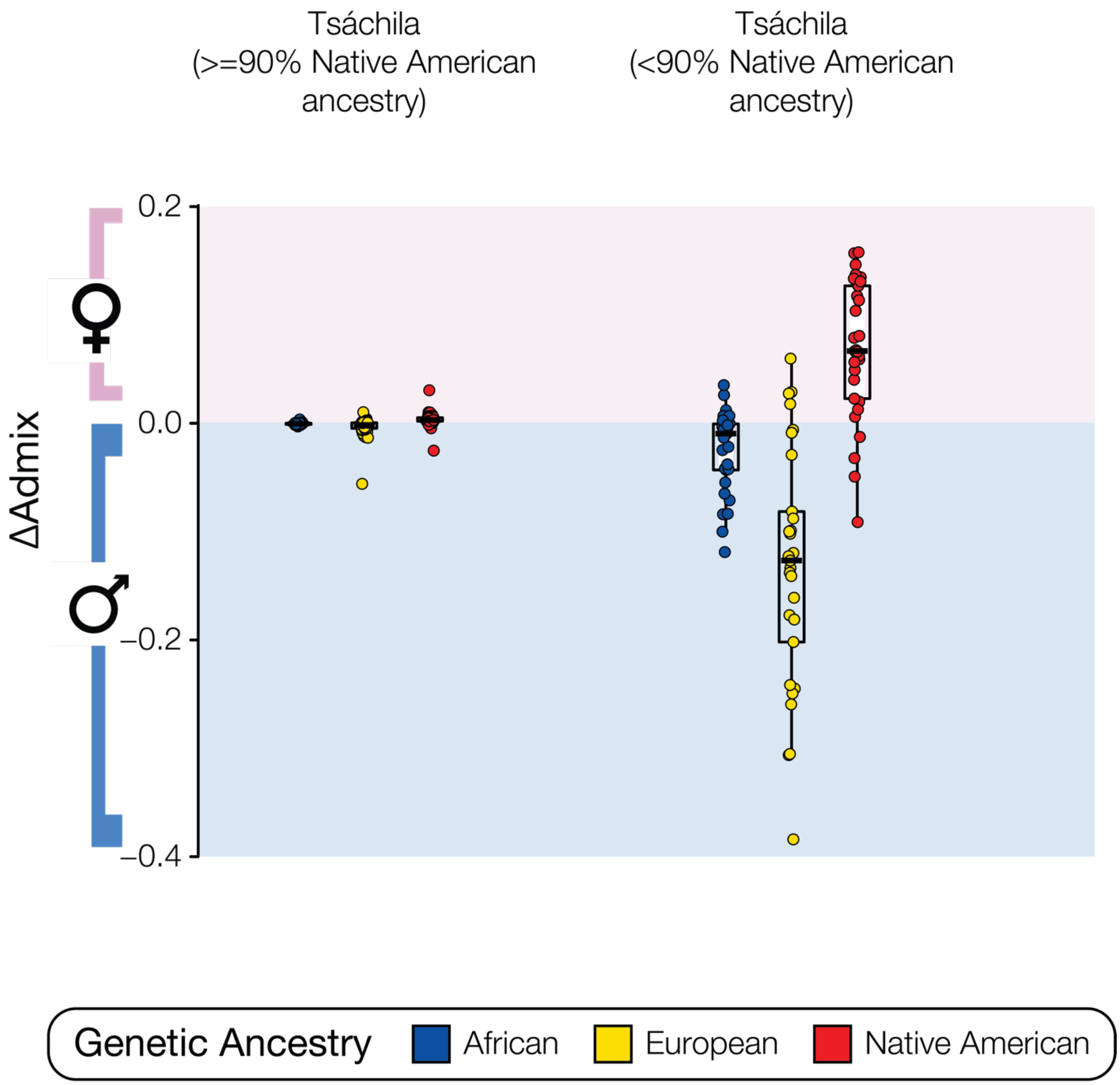

Figure S4. **Sex-biased admixture for Tsáchila subgroups.** Sex-biased ancestry proportions are shown for each of the three ancestral groups: African (blue), European (yellow), and Native American (red). Results are shown for (A) non-admixed individuals (>=90% Native American ancestry) and (B) admixed individuals (<90% Native American ancestry).

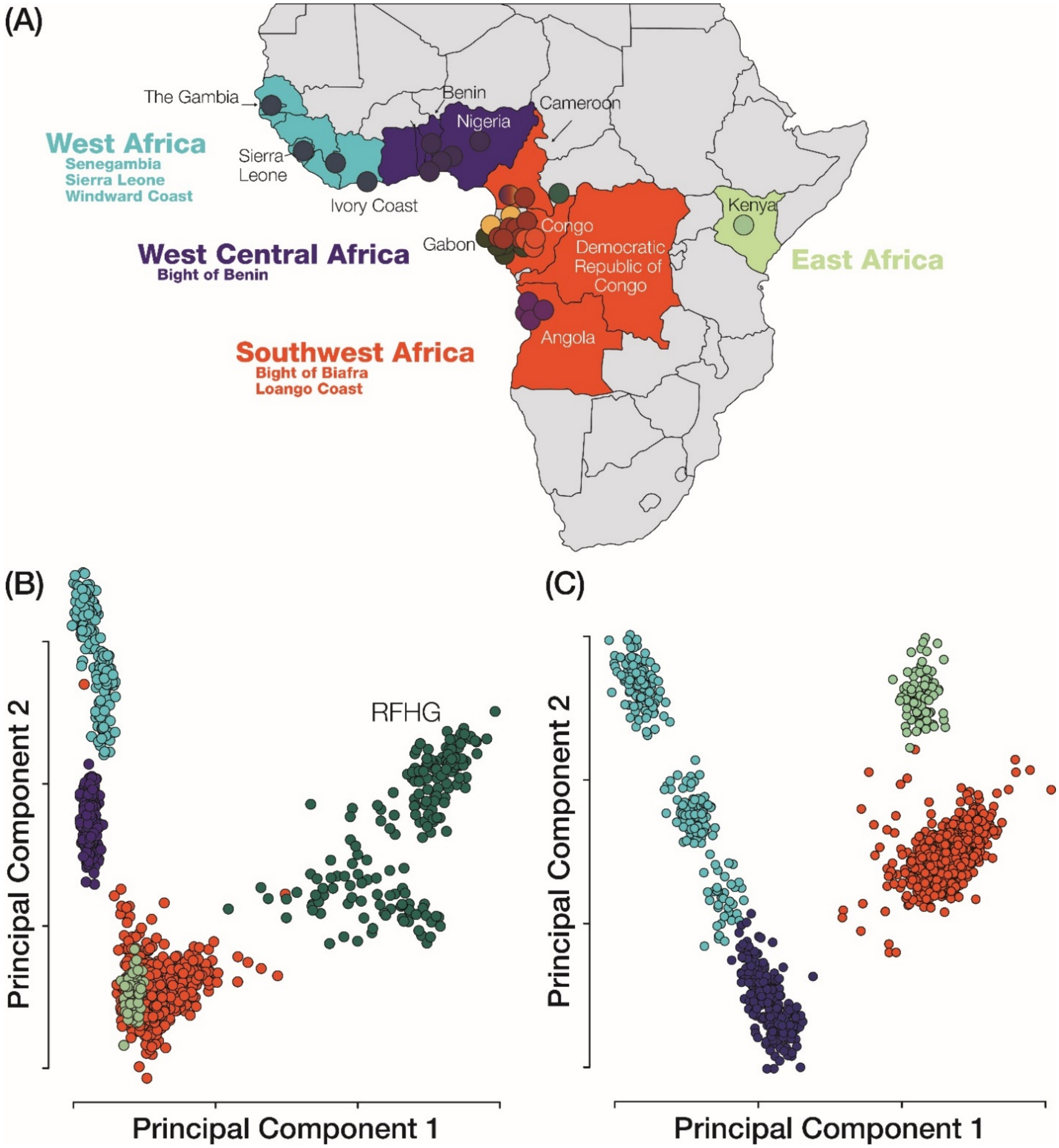

Figure S5. **African reference populations used for this study.** (A) The sampling locations of reference populations are shown, with respect to modern African countries, along with the locations of the four main geographic regions – West Africa, West Central Africa, Southwest Africa, and East Africa – and the corresponding colonial era slave trading regions along the west coast of Africa. (B&C) Principal components analyses (PCA) showing the genetic relationships among African reference population samples from the four geographic regions. Panel B includes rainforest hunter gatherer (RFHG) populations, and panel C includes only populations from the four main African regions analyzed here.

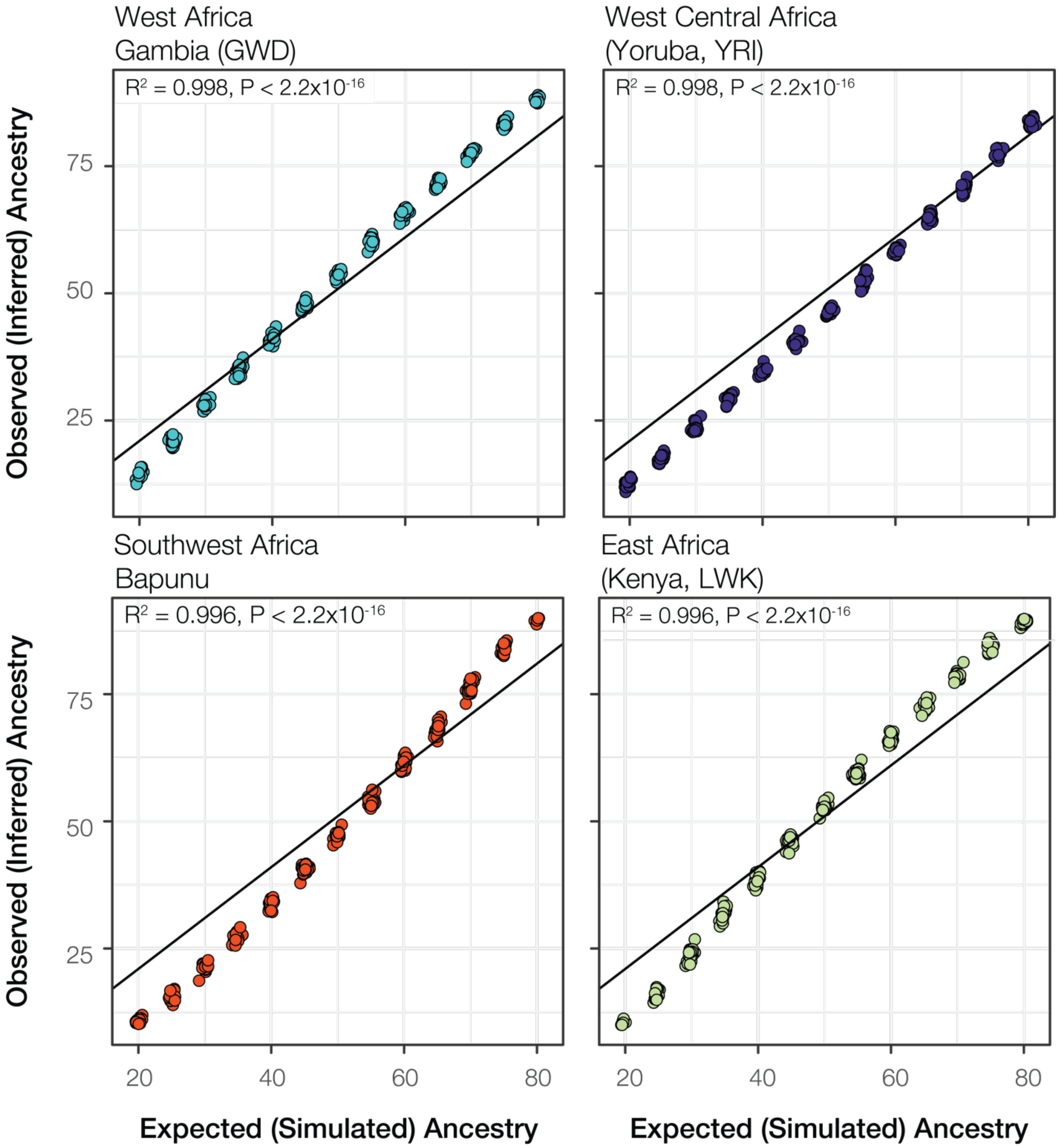

Figure S6. **Validation of African subcontinental ancestry inference.** Expected levels of ancestry based on simulated admixed genomes (x-axis) are compared to observed levels of ancestry based on NNLS method applied to simulated genomes (y-axis). Results are shown for the four main geographic regions studied here: West Africa (Gambia, GWD), West Central Africa (Yoruba, YRI), Southwest Africa (Bapunu), and East Africa (Kenya, LWK). Pearson correlation R2 and P-values are shown for each comparison.

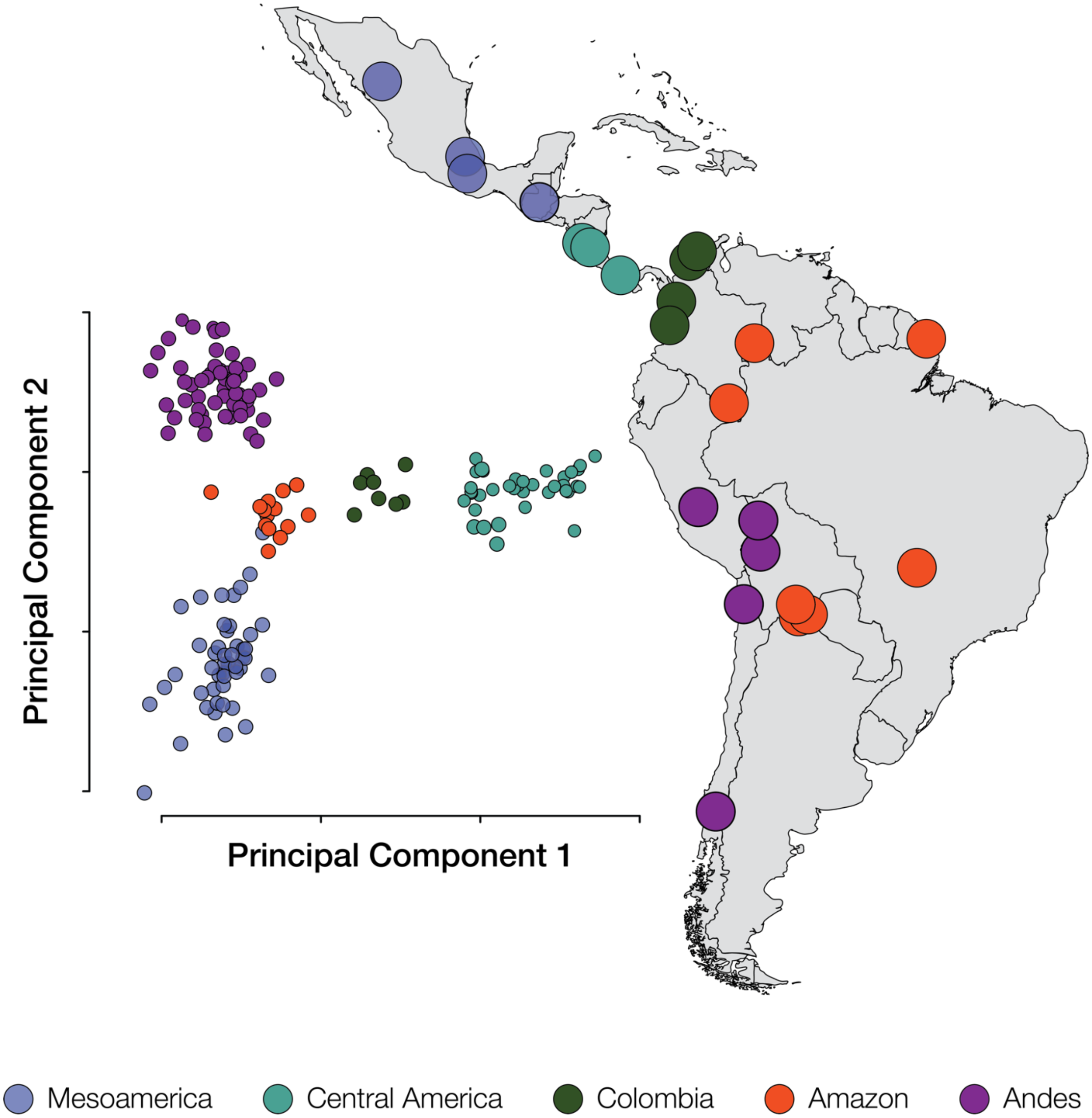

Figure S7. **Native American reference populations used for this study.** (A) The sampling locations of reference populations are shown, with respect to modern Latin American countries, along with the locations of the five main geographic/genetic groups: Mesoamerica (light blue), Central America (teal), Colombia (green), Amazon (orange), Andes (purple). (B) Principal components analysis (PCA) showing the genetic relationships among Native American reference population samples from the main groups.

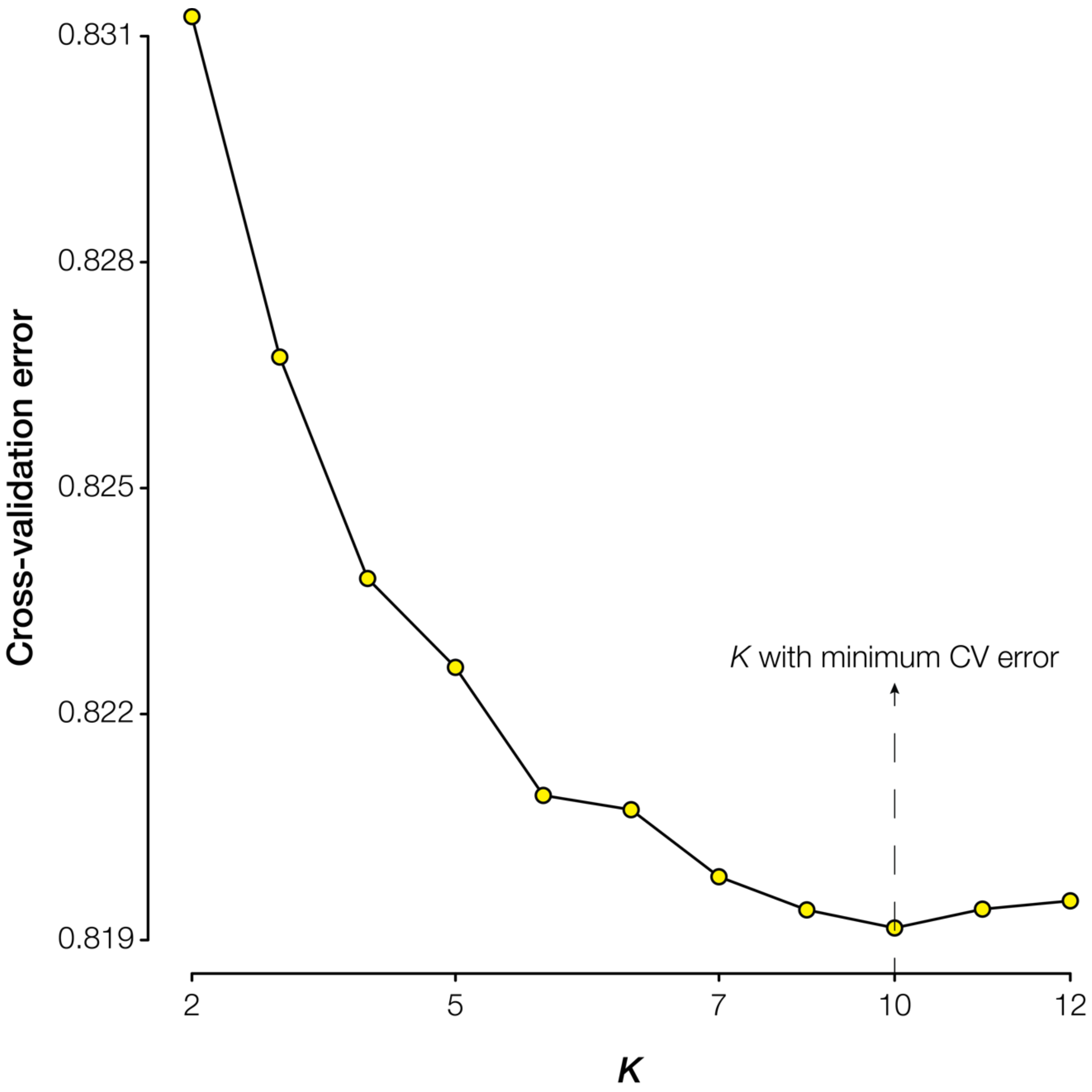

Figure S8. **Cross-validation error values (y-axis) for AMDIXTURE run over a range of ancestry component values K=2-12 on Native American ancestry.** The optimal value of K=10 was chosen for analysis Native American ancestry and admixture.

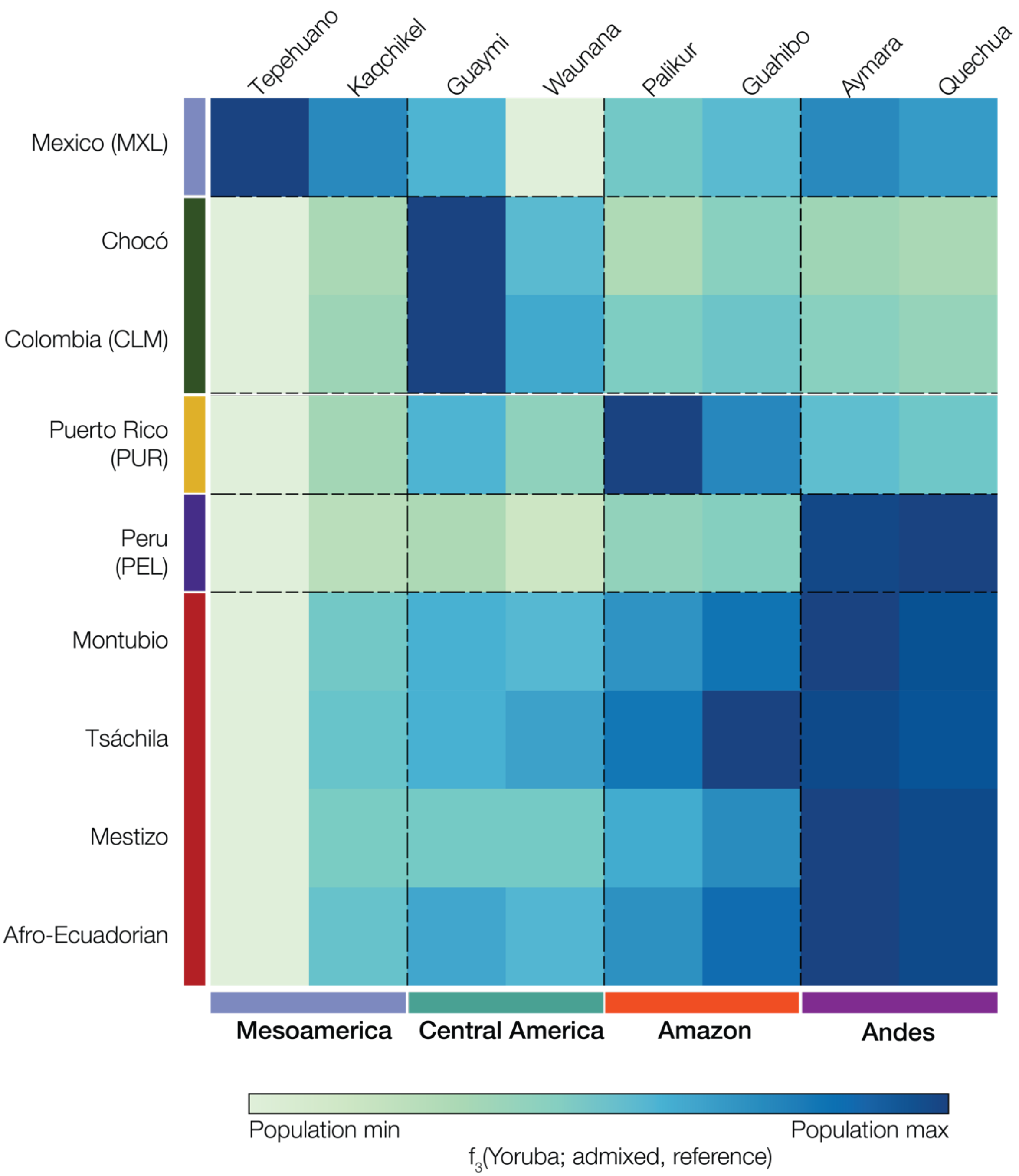

Figure S9. **Native American origins of Ecuadorian populations.** Phylogenetic similarity between the four Ecuadorian populations studied here, other admixed American populations, and Native American reference populations were assessed using the outgroup f3 statistic in the form shown. Similarity levels between admixed American populations and Native American reference populations are color coded as shown in the key.

Table S1. **Reference populations used in this study.** This table documents all the reference populations used for ancestry inference in this study – their continental and subcontinental population groups, sample size, and the technology used for genomic characterization.

#	Population	Continental group	Subcontinental group	Sample size	Genomic technology	Manuscript location	Reference
Whole genome sequence data (WGS) for continental and subcontinental ancestry inference							
1	Utah Residents (CEPH) with Northern and Western European Ancestry	European	North and Central Europe	99	WGS	Figure 1, Figure 4, Figure S2, Figure S3	1000 Genomes Project [1]
2	Finnish in Finland	European	North and Central Europe	99	WGS	Figure 4, Figure S3	1000 Genomes Project [1]
3	British in England and Scotland	European	North and Central Europe	91	WGS	Figure 1, Figure 4, Figure S2, Figure S3	1000 Genomes Project [1]
4	Toscani in Italia	European	South Europe	107	WGS	Figure 1, Figure 4, Figure S2, Figure S3	1000 Genomes Project [1]
5	Iberian Population in Spain	European	South Europe	107	WGS	Figure 1, Figure 4, Figure S2, Figure S3	1000 Genomes Project [1]
6	Gambian in Western Divisions in the Gambia	African	West Africa	113	WGS	Figure 4, Figure S3, Figure S5	1000 Genomes Project [1]
7	Mende in Sierra Leone	African	West Africa	85	WGS	Figure 1, Figure 4, Figure S2, Figure S3, Figure S5	1000 Genomes Project [1]

8	Yoruba in Ibadan, Nigeria	African	West Central Africa	108	WGS	Figure 1, Figure 4, Figure S2, Figure S3, Figure S5	1000 Genomes Project [1]
9	Esan in Nigeria	African	West Central Africa	99	WGS	Figure 1, Figure 4, Figure S2, Figure S3, Figure S5	1000 Genomes Project [1]
10	Luhya in Webuye, Kenya	African	East African	99	WGS	Figure 1, Figure 4, Figure S2, Figure S3, Figure S5	1000 Genomes Project [1]
11	Americans of African Ancestry in SW USA	Admixed American	Afro-descendant	61	WGS	Figure 1, Figure 4, Figure S2, Figure S3	1000 Genomes Project [1]
12	African Caribbeans in Barbados	Admixed American	Afro-descendant	96	WGS	Figure 1, Figure 4, Figure S2, Figure S3	1000 Genomes Project [1]
13	Puerto Ricans from Puerto Rico	Admixed American	Latin American	104	WGS	Figure 1, Figure 4, Figure 5, Figure S2, Figure S3	1000 Genomes Project [1]
14	Mexican Ancestry from Los Angeles USA	Admixed American	Latin American	64	WGS	Figure 1, Figure 4, Figure S2, Figure S3	1000 Genomes Project [1]
15	Colombians from Medellin, Colombia	Admixed American	Latin American	94	WGS	Figure 1, Figure 4, Figure 5, Figure S2, Figure S3	1000 Genomes Project [1]
16	Peruvians from Lima, Peru	Admixed American	Latin American	85	WGS	Figure 1, Figure 4, Figure 5,	1000 Genomes Project [1]

						Figure S2, Figure S3	
17	Han Chinese in Beijing, China	East Asian	Chinese	112	WGS	Figure 1, Figure S2, Figure S3	1000 Genomes Project [1]
18	Japanese in Tokyo, Japan	East Asian	Japanese	105	WGS	Figure 1, Figure S2, Figure S3	1000 Genomes Project [1]
Whole genome genotype (WGG) arrays for subcontinental ancestry inference							
African subcontinental ancestry reference samples							
19	Yacouba (Ivory Coast)	African	West Africa	20	Illumina HumanOmni Express-12 array	Figure 4, Figure S5	Patin et al., 2017 [2]
20	Ahizi (Ivory Coast)	African	West Africa	20	Illumina HumanOmni Express-12 array	Figure 4, Figure S5	Patin et al., 2017 [2]
21	Yoruba (Benin)	African	West Central Africa	20	Illumina HumanOmni Express-12 array	Figure 4, Figure S5	Patin et al., 2017 [2]
22	Bariba (Benin)	African	West Central Africa	20	Illumina HumanOmni Express-12 array	Figure 4, Figure S5	Patin et al., 2017 [2]
23	Fon (Benin)	African	West Central Africa	12	Illumina HumanOmni Express-12 array	Figure 4, Figure S5	Patin et al., 2017 [2]

24	Yaounde (Cameroon)	African	West Central Africa / Southwest Africa	39	Illumina HumanOmni Express-12 array	Figure 4, Figure S5	Patin et al., 2017 [2]
25	Kongo (Angola)	African	Southwest Africa	11	Illumina HumanOmni Express-12 array	Figure 4, Figure S5	Patin et al., 2017 [2]
26	Kimbundu (Angola)	African	Southwest Africa	18	Illumina HumanOmni Express-12 array	Figure 4, Figure S5	Patin et al., 2017 [2]
27	Ovimbundu (Angola)	African	Southwest Africa	16	Illumina HumanOmni Express-12 array	Figure 4, Figure S5	Patin et al., 2017 [2]
28	Umbundo (Angola)	African	Southwest Africa	5	Illumina HumanOmni Express-12 array	Figure 4, Figure S5	Patin et al., 2017 [2]
29	Bateke (Gabon)	African	Southwest Africa	54	Illumina HumanOmni Express-12 array	Figure 4, Figure S5	Patin et al., 2017 [2]
30	Nzebi (Gabon)	African	Southwest Africa	62	Illumina HumanOmni Express-12 array	Figure 4, Figure S5	Patin et al., 2017 [2]
31	Bapunu (Gabon)	African	Southwest Africa	53	Illumina HumanOmni	Figure 4, Figure S5	Patin et al., 2017 [2]

					Express-12 array		
32	Tsogo (Gabon)	African	Southwest Africa	65	Illumina HumanOmni Express-12 array	Figure 4, Figure S5	Patin et al., 2017 [2]
33	Eshira (Gabon)	African	Southwest Africa	41	Illumina HumanOmni Express-12 array	Figure 4, Figure S5	Patin et al., 2017 [2]
34	Galoa (Gabon)	African	Southwest Africa	50	Illumina HumanOmni Express-12 array	Figure 4, Figure S5	Patin et al., 2017 [2]
35	Orungu (Gabon)	African	Southwest Africa	22	Illumina HumanOmni Express-12 array	Figure 4, Figure S5	Patin et al., 2017 [2]
36	Duma (Gabon)	African	Southwest Africa	47	Illumina HumanOmni Express-12 array	Figure 4, Figure S5	Patin et al., 2017 [2]
37	Ndumu (Gabon)	African	Southwest Africa	38	Illumina HumanOmni Express-12 array	Figure 4, Figure S5	Patin et al., 2017 [2]
38	Obamba (Gabon)	African	Southwest Africa	46	Illumina HumanOmni Express-12 array	Figure 4, Figure S5	Patin et al., 2017 [2]

39	Benga (Gabon)	African	Southwest Africa	51	Illumina HumanOmni Express-12 array	Figure 4, Figure S5	Patin et al., 2017 [2]
40	Fang (Gabon)	African	Southwest Africa	69	Illumina HumanOmni Express-12 array	Figure 4, Figure S5	Patin et al., 2017 [2]
41	Badwee (Cameroon)	African	Southwest Africa	40	Illumina HumanOmni Express-12 array	Figure 4, Figure S5	Patin et al., 2017 [2]
42	Akele (Gabon)	African	Southwest Africa	49	Illumina HumanOmni Express-12 array	Figure 4, Figure S5	Patin et al., 2017 [2]
43	Okande (Gabon)	African	Southwest Africa	8	Illumina HumanOmni Express-12 array	Figure 4, Figure S5	Patin et al., 2017 [2]
44	Makina (Gabon)	African	Southwest Africa	45	Illumina HumanOmni Express-12 array	Figure 4, Figure S5	Patin et al., 2017 [2]
45	Bakota (Gabon)	African	Southwest Africa	56	Illumina HumanOmni Express-12 array	Figure 4, Figure S5	Patin et al., 2017 [2]
46	Bekwil (Gabon)	African	Southwest Africa	5	Illumina HumanOmni	Figure S5	Patin et al., 2017 [2]

					Express-12 array		
47	Eviya (Gabon)	African	Southwest Africa	31	Illumina HumanOmni Express-12 array	Figure S5	Patin et al., 2017 [2]
48	Shake (Gabon)	African	Southwest Africa	52	Illumina HumanOmni Express-12 array	Figure 4, Figure S5	Patin et al., 2017 [2]
49	Babongo (east) (Gabon)	African	Southwest Africa / Rain Forest Hunter Gatherer	40	Illumina HumanOmni Express-12 array	Figure 4, Figure S5	Patin et al., 2017 [2]
50	Biaka (Central African Republic)	African	Rain Forest Hunter Gatherer	20	Illumina HumanOmni Express-12 array	Figure 4, Figure S5	Patin et al., 2017 [2]
51	Baka (Cameroon)	African	Rain Forest Hunter Gatherer	117	Illumina HumanOmni Express-12 array	Figure S5	Patin et al., 2017 [2]
52	Bakoya (Gabon)	African	Rain Forest Hunter Gatherer	25	Illumina HumanOmni Express-12 array	Figure S5	Patin et al., 2017 [2]
53	Bezan (Cameroon)	African	Rain Forest Hunter Gatherer	26	Illumina HumanOmni Express-12 array	Figure S5	Patin et al., 2017 [2]

54	Mbuti (Democratic Republic of Congo)	African	Rain Forest Hunter Gatherer	13	Illumina HumanOmni Express-12 array	Figure S5	Patin et al., 2017 [2]
55	Batwa (Democratic Republic of Congo)	African	Rain Forest Hunter Gatherer	2	Illumina HumanOmni Express-12 array	Figure S5	Patin et al., 2017 [2]
**Native American subcontinental ancestry reference samples**							
56	Tepehuano (Mexico)	Native American	Mesoamerica	25	Illumina HumanHap5 50 V3.0 array	Figure 5, Figure S7, Figure S9	Reich et al., 2012 [3]
57	Mixe (Mexico)	Native American	Mesoamerica	17	Illumina 610-Quad array	Figure 5, Figure S7	Reich et al., 2012 [3]
58	Mixtec (Mexico)	Native American	Mesoamerica	5	Illumina 610-Quad array	Figure 5, Figure S7	Reich et al., 2012 [3]
59	Kaqchikel (Guatemala)	Native American	Mesoamerica	13	Illumina 610-Quad array	Figure 5, Figure S7, Figure S9	Reich et al., 2012 [3]
60	Guaymi (Costa Rica)	Native American	Central America	5	Illumina 610-Quad array	Figure 5, Figure S7, Figure S9	Reich et al., 2012 [3]
61	Teribe (Costa Rica)	Native American	Central America	3	Illumina 610-Quad array	Figure 5, Figure S7	Reich et al., 2012 [3]
62	Cabecar (Costa Rica)	Native American	Central America	31	Illumina 610-Quad array	Figure 5, Figure S7	Reich et al., 2012 [3]
63	Embera (Colombia)	Native American	Colombia	5	Illumina 610-Quad array	Figure 5, Figure S7	Reich et al., 2012 [3]
64	Kogi (Colombia)	Native American	Colombia	4	Illumina 610-Quad array	Figure S7	Reich et al., 2012 [3]
65	Chocó (Colombia)	Admixed American	Colombia	94	Illumina HumanOmni Express-24	Figure 5, Figure S7	Conley et al., 2017[4]

66	Waunana (Colombia)	Native American	Colombia	3	Illumina 610-Quad array	Figure S7, Figure S9	Reich et al., 2012 [3]
67	Wayuu (Colombia)	Native American	Colombia	11	Illumina 610-Quad array	Figure S7	Reich et al., 2012 [3]
68	Piapoco (Colombia)	Native American	Amazon	7	Illumina 610-Quad array	Figure 5, Figure S7	Reich et al., 2012 [3]
69	Guahibo (Colombia)	Native American	Amazon	6	Illumina 610-Quad array	Figure 5, Figure S7, Figure S9	Reich et al., 2012 [3]
70	Guarani (Paraguay & Argentina)	Native American	Amazon	6	Illumina 610-Quad array	Figure 5, Figure S7	Reich et al., 2012 [3]
71	Palikur (Guiana)	Native American	Amazon	3	Illumina 610-Quad array	Figure 5, Figure S7, Figure S9	Reich et al., 2012 [3]
72	Ticuna (Colombia)	Native American	Amazon	6	Illumina 610-Quad array	Figure S7	Reich et al., 2012 [3]
73	Toba (Argentina)	Native American	Amazon	4	Illumina 610-Quad array	Figure S7	Reich et al., 2012 [3]
74	Wichi (Argentina)	Native American	Amazon	5	Illumina 610-Quad array	Figure S7	Reich et al., 2012 [3]
75	Aymara (Bolivia & Chile)	Native American	Andes	23	Illumina 610-Quad array	Figure 5, Figure S7, Figure S9	Reich et al., 2012 [3]
76	Inga (Colombia)	Native American	Andes	9	Illumina 610-Quad array	Figure S7	Reich et al., 2012 [3]
77	Quechua (Bolivia & Peru)	Native American	Andes	40	Illumina 610-Quad array	Figure 5, Figure S7, Figure S9	Reich et al., 2012 [3]
78	Hulliche (Chile)	Native American	Andes	4	Illumina 610-Quad array	Figure S7	Reich et al., 2012 [3]

WGS – Whole Genome Sequencing

References:

1. Genomes Project C, Auton A, Brooks LD, et al. A global reference for human genetic variation. *Nature* 2015; **526**(7571): 68-74.

2. Patin E, Lopez M, Grollemund R, et al. Dispersals and genetic adaptation of Bantu-speaking populations in Africa and North America. *Science* 2017; **356**(6337): 543-6.

3. Reich D, Patterson N, Campbell D, et al. Reconstructing Native American population history. *Nature* 2012; **488**(7411): 370-4.

4. Conley AB, Rishishwar L, Norris ET, et al. A Comparative Analysis of Genetic Ancestry and Admixture in the Colombian Populations of Choco and Medellin. *G3 (Bethesda)* 2017; **7**(10): 3435-47.
