# Supplementary material for: Genetic ancestry and ethnic identity in Ecuador
Source: HGG Adv. 2021 Aug 20;2(4):100050. doi: 10.1016/j.xhgg.2021.100050 (PMC8756502; doi:10.1016/j.xhgg.2021.100050)
Supplement: Data S1. Informed consent form [file mmc2.pdf]

Informed Consent Form

Project objective: to determine the existing genetic relationships between the ethnic population groups of Ecuador. Population genetics is the study of the forces that alter the genetic makeup of a species. It deals with micro-evolutionary change mechanisms: mutation, natural selection, gene flow, and gene drift.
Voluntary participation: You can freely choose whether or not you want to participate in this study. There is no type of penalty if you do not wish to participate or wish to withdraw from the study in any of this project's phases. We do not ask for any explanation for your withdrawal. You must understand what is required of you in this project, so we are always ready to answer your questions or clarify your doubts; In addition to providing detailed information on the phase of the project where we are, please ask the interviewer or contact one of the people named at the end of this sheet. Your participation in this project has no cost.
Blood collection: A healthcare professional punctures one of your fingers and the blood are collected on an FTA® Whatman card.
Confidentiality: All information you provide we kept entirely confidential, and we use it only for research purposes without any connection to your name. We share the general results of the research only with the scientific community in general.
Risks and benefits: Because the method is only to take a blood sample (conventional method of diagnosis), there are no risks to your health. The benefit of their participation is to be able to establish the mechanisms of micro-evolutionary change: mutation, natural selection, gene flow, and gene drift. This research will help the scientific community to understand the genetic relationships that exist among modern Ecuadorians.

Name of person taking the sample: ____________________

Location: ____________________

Canton: ____________________

Province: ____________________

Informed Consent

I,____

ID number____

I come freely, voluntarily, altruistically to donate a sample of my blood obtained from one of my fingers, and that will be collected on an FTA card, for subsequent DNA analysis for anthropological, population and research purposes genetics genomics. The sample does NOT be used for diagnostic or individual treatment purposes. The samples obtained are anonymous, properly archived, and the study's genetic data are confidential. The donated sample may be used for all types of DNA analysis for scientific research purposes and may not be used for commercial or other purposes not authorized in this consent. I also declare that I support scientific research as a mechanism for social development and advancement of knowledge, so my participation in this project is selfless and non-profit. I authorize the study researchers to use the donated sample in the proposed research or others derived from the knowledge obtained through it.

I declare that I have read this document, and I fully agree with it.

Sign:

Date:

Donor

Family name	
First name	
Age	
Sex	
Ethnic group	
Language	
Place of birth	
Place of residence	
Health condition	

Father		Mother	
Family names		Family names	
Place of birth		Place of birth	
Place of residence		Place of residence	
Ethnic group		Ethnic group	
Health condition		Health condition	
Language		Language	

Paternal grandfather		Paternal grandmother	
Family names		Family names	
Place of birth		Place of birth	
Place of residence		Place of residence	
Ethnic group		Ethnic group	
Health condition		Health condition	
Language		Language	

Maternal grandfather		Maternal grandmother	
Family names		Family names	
Place of birth		Place of birth	
Place of residence		Place of residence	
Ethnic group		Ethnic group	
Health condition		Health condition	
Language		Language	
