# Supplementary material for: Genetic ancestry and ethnic identity in Ecuador
Source: HGG Adv. 2021 Aug 20;2(4):100050. doi: 10.1016/j.xhgg.2021.100050 (PMC8756502; doi:10.1016/j.xhgg.2021.100050)
Supplement: Document S2. Article plus supplemental information [file mmc3.pdf]

HGG
Advances

ARTICLE

Genetic ancestry and ethnic identity in Ecuador

Shashwat Deepali Nagar,[1,2] Andrew B. Conley,[2,3,4] Aroon T. Chande,[1,2,3] Lavanya Rishishwar,[2,3,4] Shivam Sharma,[4] Leonardo Mariño-Ramírez,[2,4] Gabriela Aguinaga-Romero,[5] Fabricio González-Andrade,[5,6,*] and I. King Jordan[1,2,3,6,*]

Summary

We investigated the ancestral origins of four Ecuadorian ethnic groups—Afro-Ecuadorian, Mestizo, Montubio, and the Indigenous Tsáchila—in an effort to gain insight on the relationship between ancestry, culture, and the formation of ethnic identities in Latin America. The observed patterns of genetic ancestry are largely concordant with ethnic identities and historical records of conquest and colonization in Ecuador. Nevertheless, a number of exceptional findings highlight the complex relationship between genetic ancestry and ethnicity in Ecuador. Afro-Ecuadorians show far less African ancestry, and the highest levels of Native American ancestry, seen for any Afro-descendant population in the Americas. Mestizos in Ecuador show high levels of Native American ancestry, with substantially less European ancestry, despite the relatively low Indigenous population in the country. The recently recognized Montubio ethnic group is highly admixed, with substantial contributions from all three continental ancestries. The Tsáchila show two distinct ancestry subgroups, with most individuals showing almost exclusively Native American ancestry and a smaller group showing a Mestizo characteristic pattern. Considered together with historical data and sociological studies, our results indicate the extent to which ancestry and culture interact, often in unexpected ways, to shape ethnic identity in Ecuador.

Introduction

The South American country of Ecuador is home to a multiethnic society that emerged from contact among the numerous Indigenous communities that inhabited the region for millennia, European colonizers (Spanish conquistadors and mercantile immigrants), and enslaved Africans brought to the New World by force.[1] Similar to other Latin American nation-states, interactions among these groups over the last five centuries have led to the consolidation of ethnic identities that were initially grounded in varying degrees of admixture. Examining the genetic ancestry of different groups in Ecuador can give us insight into how ethnic identities, both old and new, are shaped and have possibly changed from the original *castas* definitions instituted during colonial times.[2,3] Here, we investigate the ancestral origins of four officially recognized Ecuadorian ethnic groups: Afro-Ecuadorian, the majority Mestizo ethnic group, the newly recognized Montubio ethnic group, and the Indigenous Tsáchila. This is the first genetic study of the Montubio and Tsáchila populations.

The Ecuadorian census defines five major ethnic groups: Afro-Ecuadorian, Indigenous, Blanco, Mestizo, and Montubio.[4,5] Mestizos—historically defined as the descendants of Europeans and Indigenous Americans—constitute the largest part (71.9%) of the Ecuadorian population according to the current census (2010). Individuals who self-identify as Mestizo live primarily in urban areas and are native speakers of Spanish. Along with Mestizos, Ecuador has fourteen distinct Indigenous groups, inhabiting different parts of the country. Ecuadorians who identify as Indigenous make up 7.0% of the population. In this report, we focused on the relatively under-studied Tsáchila Indigenous group, who are the native inhabitants of the Santo Domingo province of Ecuador and are speakers of the Tsáfiqui language. Montubios were officially recognized as a distinct ethnocultural group by the government of Ecuador in 2001 and represent 7.4% of the total population. Recognition of the Montubio as a distinct ethnic group followed years of struggle, including a protracted hunger strike that drew widespread attention to their cause.[6] Montubios are thought to descend from Indigenous groups who traditionally inhabited the coastal regions of Ecuador and later admixed with Spanish settlers and enslaved or freed Africans, starting in the colonial era.[7–9] The extent of ancestral contributions from each of these three groups to the modern Montubios is an open question. Afro-Ecuadorians, who live primarily in the provinces of Esmeraldas and Guayas, make up 7.2% of the Ecuadorian population. The presence of Afro-Ecuadorians in Esmeraldas dates to 1553, when a group of 23 Africans escaped from a stranded slave ship and mixed with the local Indigenous groups to establish an autonomous community. From these small beginnings, the region continued to receive an influx of escaped slaves, from both Ecuador and Colombia, giving rise to a large and independent Afro-descendant population.[10,11]

Nation building in colonial-era Latin America was explicitly aware of, and informed by, notions of race and

[1]School of Biological Sciences, Georgia Institute of Technology, Atlanta, GA, USA; [2]PanAmerican Bioinformatics Institute, Cali, Valle del Cauca, Colombia; [3]IHRC-Georgia Tech Applied Bioinformatics Laboratory, Atlanta, GA, USA; [4]National Institute on Minority Health and Health Disparities, National Institutes of Health, Bethesda, MD, USA; [5]Faculty of Medical Sciences, Central University of Ecuador, Quito, Ecuador
[6]These authors contributed equally
*Correspondence: fabriciogonzaleza@gmail.com (F.G.-A.), king.jordan@biology.gatech.edu (I.K.J.)
https://doi.org/10.1016/j.xhgg.2021.100050.
© 2021 The Authors. This is an open access article under the CC BY license (http://creativecommons.org/licenses/by/4.0/).

ancestry. An emphasis was placed on the delineation of new racial (ethnic) groups formed by various combinations of the three continental ancestry groups that came together in the New World: African, European, and Native American. This movement reached its apogee with the Spanish *Sistema de Castas* (Caste System).[2,3] Under this racialized classification scheme, numerous groups were defined by specific combinations of admixture, often in a very granular way across multiple generations. The *Sistema de Castas* was inherently hierarchical, with European (Spanish) ancestry at the top and Native American or African ancestry at the bottom. High levels of Spanish ancestry were almost always associated with higher social status. The related concept of *mestizaje* refers to the underlying process of racial and cultural mixing, also with an implicit preference for Spanish ancestry and culture.[12–15]

In Ecuador, the process of *mestizaje* played out in a very particular way, whereby the initial ancestry-based definition of Mestizo slowly gave way to a more culture-based definition that was tied to language, education, and social status. Given the conflation of ancestry, culture, and social status with ethnicity in Ecuador, the ancestral origins and makeup of different ethnic groups have been obscured. Genetic studies, considered in the context of this unique history, can shed light on the extent to which ancestry does or does not contribute to Ecuadorians' ethnic identities. Indeed, there is a growing interest among Ecuadorians to better understand the ancestral composition of these socially constructed ethnic groups, particularly with respect to historically marginalized Indigenous and African identities.[16–18]

Previous studies have characterized the genetic origins of the different ethnic groups in Ecuador,[16,17,19] but they have only described a small part of the vivid landscape of genetic diversity in Ecuador. These efforts were limited either by (1) the populations sampled, which often included only Mestizos or did not specify the Ecuadorian sub-population; or (2) their use of limited numbers of Ancestry Informative Markers (AIMs), which do not allow for deep characterization of genetic ancestry. In this study, we use genome-wide variant data from four distinct Ecuadorian populations, which allowed us to infer fine-scale population structure and the ancestral origins of ethnic groups that collectively represent most of the Ecuadorian population. In addition to the widely studied majority Mestizo population, we characterized the genetic ancestry for previously understudied and historically neglected Afro-Ecuadorian, Montubio, and Tsáchila minority groups.

Material and methods

Population terminology

The terminology used to describe the populations studied here is intended to distinguish ethnicity from genetic ancestry. For ethnicity, we use the names of the officially recognized Ecuadorian ethnic groups following the 2010 census: Afro-Ecuadorian (translated from the Spanish *Afroecautoriano*), Indigenous (translated from the Spanish *Indigena*), Blanco, Mestizo, and Montubio.[4,5] We use the broad term Afro-descendant (translated from the Spanish *afrodescendiente*), which is widely used in Latin America to refer to the descendants of African people who arrived in the Americas via the trafficking of enslaved persons during the colonial era.[20] While many individuals in Latin America have some degree of African ancestry, Afro-descendants identify as having direct ancestral and cultural connections to Africa. We use the officially recognized name Tsáchila for the Indigenous ethnic subgroup studied here. We use the broad term Indigenous to describe the original, native inhabitants of the Americas, including but not limited to Ecuador. For genetic ancestry, we use continental ancestry group labels—African, European, and Native American—following conventions of the scientific literature on genetic ancestry.[21–24] The presence of Native American ancestry in any individual does not necessarily imply tribal affiliation or identity with a specific Indigenous group.

Donor sample collection

DNA samples were characterized for 300 sample donors from four Ecuadorian ethnic groups across seven locations: Afro-Ecuadorian (91), Mestizo (35), Montubio (82), and Tsáchila (92). Afro-Ecuadorian samples were taken in the city of Esmeraldas in Esmeraldas province and in the Chota Valley, located between provinces of Imbabura and Carchi. Montubio samples were taken in the cities of Portoviejo, Jipijapa, and Chone in the Manabí province. Mestizo samples were taken in Quito, Pichincha. Tsáchila samples were taken in Santo Domingo de los Colorados of the province of Santo Domingo de los Tsáchilas. Blood samples were obtained from donors by the finger-prick method and collected on FTA cards (GE Healthcare Life Sciences). Donors provided demographic information for themselves, their parents, and their grandparents: surnames, place of birth, place of residence, ethnic self-identity, and spoken language. The same technician was responsible for collecting all donor blood samples and demographic information. Donor blood samples and demographic data were collected in accordance with the ethical standards of the 1964 Declaration of Helsinki and its later amendments. All samples were provided voluntarily, de-identified, and securely archived. All donors approved and signed the Informed Consent Form (Data S1), and donor sampling was approved by Translational Medicine Unit of Faculty of Medical Sciences at Central University of Ecuador, in Quito, Ecuador. Genome analysis of the samples was also approved by the Institutional Review Board of the Georgia Institute of Technology.

Genome characterization and analysis

DNA was extracted from FTA card blood spots using QIAGEN's DNeasy Blood & Tissue Kits. Genome-wide genotyping was performed for 22 Mestizo samples using the Multi-Ethnic Global Array[25] (MEGA) to characterize ~1.7 million variants. The remaining 278 samples were characterized using the Illumina Global Screening Array (GSA) to characterize ~690,000 variants. Genome-wide genotype data from Ecuadorian populations were merged with different reference panels for characterizing (1) global and local continental ancestry, and (2) African, European, and Native American subcontinental ancestry, yielding 3 different genome-wide variant datasets for subsequent analysis. The workflow for genotype data harmonization and analysis is illustrated in Figure S1, and the reference populations used in this study are listed in Table S1. Genome-wide genotype data from Ecuador are available upon request from the corresponding authors.

For characterizing global and local continental ancestry, the Ecuadorian genome-wide genotype data were merged and harmonized with whole genome sequence data from global reference populations representing four continental population groups—African, East Asian, European, and Native American—characterized as part of the 1000 Genomes Project,[26] using PLINK v.1.90[27] and bespoke scripts. The genetic variant data from the Ecuadorian samples and the reference samples were merged to include variants that were present in both datasets with a missingness and minor allele frequency filters of 5% and 1%, respectively. Variant strand flips and identifier inconsistencies were corrected as needed. The merged and harmonized dataset contained 371,355 genome-wide variants. The PLINK implementation of the KING algorithm was used to test for kinship among individuals from the harmonized dataset and to exclude one member of each pair of samples with a kinship coefficient > 0.25.[28] Next, the merged dataset of unrelated individuals was pruned for linkage disequilibrium (LD) using the "-indep" command in PLINK 1.9 with a window size of 50 kb, a step size of 5 variants, and a variant inflation factor (VIF) threshold of 2. The program ShapeIT version 2.r837[29] was used to phase the merged and harmonized variant dataset. Phasing was performed on all individuals at the same time and without reference haplotypes. Each chromosome was phased separately, and the X chromosome was phased using the "-X" flag. Together, these steps yield a final variant dataset for continental ancestry inference covering 220,009 genomic sites for 275 Ecuadorian samples and 1,728 reference population samples.

The final continental ancestry variant dataset was further harmonized with two additional reference panels for characterizing subcontinental ancestry. We separately merged the final variant dataset with (1) 1,235 African genomes across 37 additional African reference populations,[30] and (2) 251 Native American genomes across 23 Native American reference populations[31] to yield an African-harmonized dataset and a Native American-harmonized dataset (Table S1). The African-harmonized variant dataset covers 157,746 genomic sites, and the Native American-harmonized variant dataset covers 56,937 genomic sites. For European subcontinental affinity characterization, the final variant dataset was used directly, since the 1000 Genomes Project contains five European reference populations, covering all of the main source regions for immigration to the Americas. Distinct African and Native American reference panels, and the resulting merged datasets, were created owing to the fact that African and Native American reference panels were characterized on different genotyping technologies, often leading to a small overlap of genetic variants after harmonization.

Ancestry and admixture analysis

Principal component analysis (PCA) of the final continental ancestry dataset was performed using PLINK using the "-pca" option, and the first two PCs for all samples were plotted using the ggplot2 package[32] in R v.3.5.1.[33] ADMIXTURE v.1.30[34] was used to characterize samples' genome-wide ancestry fractions for four continental ancestral population components—African, East Asian, European, and Native American—using 1000 Genomes Project reference population samples (Table S1). ADMIXTURE was run in the unsupervised mode with default settings and $K = 4$. Admixture entropy values are measured as the Shannon's entropy (S) of the four ancestry component fractions: $S = -\sum_{i=1}^{4} p_i \log(p_i)$, where p_i is the population fraction for ancestry component i.

A modified version of RFMix[22,35] was used to characterize local ancestry patterns for the three main continental ancestral population components observed in the Ecuador samples—African, European, and Native American—on the final dataset. RFMix was run using African and European reference population samples from the 1000 Genomes Project, and samples from Peru were used as a surrogate for Native American ancestry. RFMix was run for 12 generations in the "PopPhased" mode with a minimum node size of five, and the "-use-reference-panels-in-EM" for two rounds of expectation maximization (EM), to assign continental ancestry for haplotypes genome-wide. Haplotype ancestry assignments were made for regions where the RFMix ancestral certainty was at least 95%.

The RFMix ancestry assignments were used to generate masked genomes, each of which only contain haplotypes from one of the three main continental ancestry groups, for subsequent subcontinental ancestry inference using either the program Chromopainter version 2[36] (African and European) or ADMIXTURE (Native American). Chromopainter was run on each of the African and European ancestry-specific masked genomes separately for each individual, comparing to either the corresponding harmonized variant dataset (African) or the final variant dataset (European). Non-negative least-squares (NNLS) was used to convert Chromopainter output painting vectors to percent ancestry estimates using the R package nnls version 1.4,[37] as we described previously.[22]

Subcontinental ancestry inference validation was performed by generating simulated admixed genomes via Monte Carlo simulation of reference population haplotypes. For the simulation of admixed genomes, reference populations were divided into training and validation samples. Training samples were used to simulate admixed genomes, and validation samples were used for subcontinental ancestry inference on the simulated admixed genomes. Simulated admixed genomes were generated using genome-wide haplotype boundaries, defined using interpolated genetic map positions based on the 1000 Genomes Project, and haplotypes were randomly selected from reference population training samples across 20%–80% admixture proportions in 5% increments, generating 20 simulated genomes for each 5% increment. For sets of simulated admixed genomes, the simulated (expected) ancestry proportions were compared to the observed ancestry proportions inferred via the NNLS approach, using Pearson correlation, to estimate the accuracy of the NNLS method for subcontinental ancestry inference. Simulated admixed genomes were generated and validated in this way for African subcontinental ancestry inference (West Africa, West Central Africa, Southwest Africa, and East Africa) and European subcontinental ancestry inference (North/Central Europe and South Europe).

Subcontinental ancestry for Native American populations was discerned using Native American masked genomes with the Native American-harmonized dataset using ADMIXTURE run in unsupervised mode, with values of K ranging from 2–12. Cross-validation error values were calculated for each value of K and used to select the optimal value of $K = 10$ for subsequent analysis. Native American subcontinental ancestry was characterized using ADMIXTURE, because haplotypes in Native American populations are quite distinct, owing to very high levels of population structure among these populations,[38] and the Chromopainter approach is not suited for these data. Phylogenetic analysis of Native American reference populations, and the Native American ancestry component of the Ecuadorian populations, was performed by calculating pairwise population F_{ST} values with smartpca from the EIGENSOFT package version 7.2.1.[39] Pairwise F_{ST} values were used to make a neighbor-joining tree[40] with the program MEGA6,[41] and bootstrap analysis was performed using prop.part and part.clades tools of the Ape package[42] version 5.4. The outgroup f_3 statistic for Native

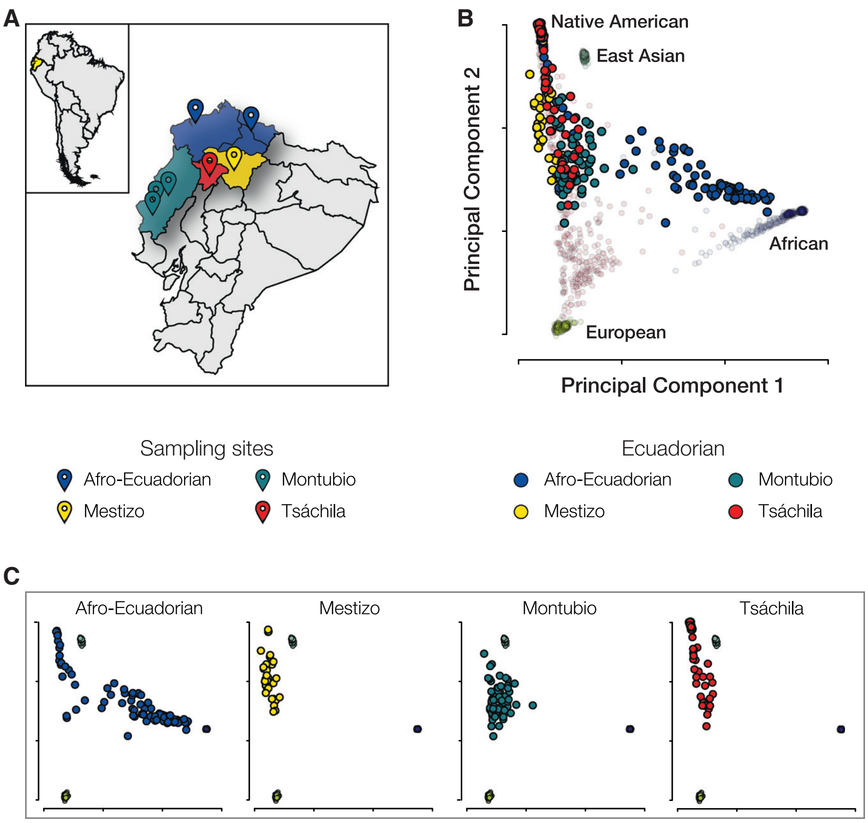

Figure 1. Continental genetic ancestry for Ecuadorian ethnic groups
(A) Locations of sampling sites for the four Ecuadorian ethnic groups characterized here: Afro-Ecuadorian (blue), Mestizo (yellow), Montubio (teal), and Tsáchila (red).
(B) Principal component analysis (PCA) of the genomic relationship matrix for the four Ecuadorian populations compared to reference populations from Africa, the Americas, East Asia, and Europe.
(C) PCA results shown separately for each of the four Ecuadorian ethnic groups.

American ancestry component of the Ecuadorian populations was computed as $f_3(Yoruba; Ecuadorian, Native\ American\ reference)$ using the masked Native American haplotypes with the program AdmixTools version 7.0.1.

Admixture timing analysis

The TRACTS program[43,44] was used to infer the timing of admixture events in the admixed populations from the ancestry-specific haplotypes (i.e., ancestry tracts) defined by RFMix. For the Afro-Ecuadorian and Montubio three-way admixed populations, three possible orderings of admixture were evaluated with TRACTS: (1) European, Native American, and African; (2) European, African, and Native American; and (3) African, Native American, and European. For the Mestizo two-way admixed population, two possible orderings of admixture were evaluated with TRACTS: (1) European and Native American, and (2) Native American and European. For each ordering, TRACTS evaluated possible admixture timing from 14 to six generations ago, in 1,000 bootstrap attempts. From the bootstrap attempts, the most likely series of admixture events was chosen to represent the population.

Sex-biased admixture inference

Sex bias in admixture for the different Ecuadorian population groups was inferred by comparing the ancestral composition of the X chromosome to the autosomes as previously described.[21,45] For each Ecuadorian sample, the normalized difference between each ancestral component for the X chromosome versus the autosomes ($\Delta Admix$) is defined as:

$$\Delta Admix = F_{anc\ \ total} \times \frac{F_{anc,X} - F_{anc,auto}}{F_{anc,X} + F_{anc,auto}},$$

where $F_{anc,total}$, $F_{anc,X}$, and $F_{anc,auto}$ refer to genome-wide, X chromosome, and autosomal ancestry fractions, respectively.

Results

Continental genetic ancestry

Individuals from four Ecuadorian ethnic groups were sampled from seven sites around the country: Afro-Ecuadorian from Esmereldas and the Chota Valley, Mestizo from Quito, Montubio from Manabí, and Tsáchila from Santo Domingo de los Colorados (Figure 1A). Genome-wide genotypes for individual sample donors were characterized and compared to global reference populations from Africa, the Americas, Asia, and Europe. PCA was used to visualize the genetic relatedness among individuals from the Ecuadorian and reference populations (Figures 1B and 1C; Figure S2). African, European, and Native American continental ancestry groups are clearly separated as three poles of diversity on the two-dimensional PCA plot, and Ecuadorian populations are clustered between the three continental ancestry groups. Most Afro-Ecuadorians fall close to the African pole, as would be expected; however, a number of Afro-Ecuadorians cluster very closely with the Native American pole. Mestizo individuals fall along the European-Native American axis, with little apparent African admixture. It should be noted that Mestizos sampled from Ecuador fall much closer to the Native American than the European pole, pointing to a relatively high Native American contribution to their genetic ancestry. The Montubios seem to lie mostly along the European-Native American axis, with some of them extending toward the African pole, indicative of low levels of African admixture in this population. Finally, most of the Tsáchila individuals cluster tightly at the Native American pole, but some individuals extend toward the European pole, suggesting similar levels of admixture compared to what is observed for some Mestizos.

The program ADMIXTURE was used to quantify the levels of continental ancestry—African, East Asian,

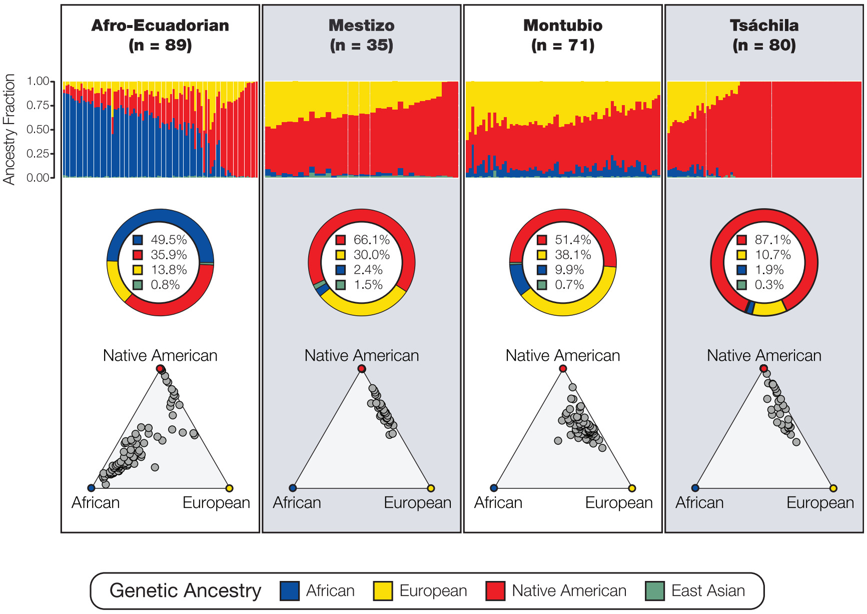

Figure 2. Continental ancestry and admixture for Ecuadorian ethnic groups
Top row: ADMIXTURE plots showing continental ancestry fractions for individuals from the four Ecuadorian ethnic groups. Each bar in the plot represents an individual sample, and individuals' ancestry components are shown as African (blue), East Asian (green), European (yellow), and Native American (red). Middle row: average ancestry percentages for each ethnic group. Bottom row: ternary plots highlighting the distribution of different proportions of the three main ancestry components—African, European, and Native American—in each Ecuadorian group.

European, and Native American—for the four Ecuadorian populations compared to other admixed American populations (Figure 2; Figure S3). On average, Afro-Ecuadorians have 49.5% African genetic ancestry, 35.9% Native American ancestry, 13.8% European ancestry, and 0.8% East Asian ancestry. Some of the individuals who identify as Afro-Ecuadorian have high fractions of African ancestry, while others show almost completely Native American ancestry. The Afro-Ecuadorian population shows the highest overall variance in ancestry components for the Ecuadorian groups. Mestizo individuals have mostly Native American ancestry (66.1%), with some European admixture (30.0%), and small amounts of African (2.4%) and East Asian genetic ancestry (1.5%). The Montubios show primarily Native American ancestry (51.4%), followed by substantial European ancestry (38.1%), along with lower levels of African ancestry (9.9%) and very little East Asian ancestry (0.7%). It should be noted that the Montubio population has 4.1 and 5.2 times as much African ancestry when compared to the Mestizo or Tsáchila populations, respectively. Tsáchila individuals in the data have high overall Native American ancestry (87.1%), followed by European (10.7%), African (1.9%), and East Asian ancestry (0.3%). The Tsáchila population includes two distinct groups of individuals, one group with almost entirely Native American ancestry and a second admixed group. The overall continental genetic ancestry fractions for the Ecuadorian populations, compared to six other admixed American populations, are summarized in Table 1.

Timing and sex bias for continental admixture

Continental ancestry was inferred at the local level by assigning genome-wide haplotype origins corresponding to the three major ancestry components of the Ecuadorian populations—African, European, and Native American—using a modified version of the program RFMix.[35] East Asian ancestry was not considered for local ancestry inference owing to the very low levels observed for the Ecuadorian populations studied here. The patterns of local ancestry were then used to infer the timing of continental admixture for the Ecuadorian ethnic groups based on the size distributions of ancestry-specific haplotypes using the program TRACTS. This analysis relies on the fact that the sizes of ancestry-specific haplotypes in admixed genomes decay over time owing to recombination. Three-way admixture models were run with TRACTS for the Afro-Ecuadorian and Montubio populations, and a two-way admixture model was run for the Mestizo population. The Afro-Ecuadorian population shows evidence of initial rounds of admixture between Native American and European ancestry components 10 and 9 generations ago, followed by two more recent pulses of African admixture 6 and 5 generations ago (Figure 3A). The Montubio population shows primarily Native American and European admixture 11 and 10 generations ago, with a much smaller round of African admixture occurring 9 and 8 generations ago (Figure 3B). The Mestizo population shows two pulses of Native American and European admixture 10 and 9 generations ago (Figure 3C).

The chromosomal distributions of ancestry-specific haplotypes can also be used to evaluate sex-biased patterns of continental admixture. Since X chromosomes spend twice as much time along the female lineage, compared to autosomes, excess continental ancestry on the X chromosome indicates female-biased admixture, whereas excess continental ancestry on the autosomes points to male-biased admixture. We previously developed the ΔAdmix parameter, which quantifies differences in ancestry between the X chromosome and the autosomes, to test for sex-biased ancestry in admixed American populations.[45] The four Ecuadorian ethnic groups studied here all show evidence of sex-biased admixture, each with its own characteristic pattern (Figure 3D). However, all four of the populations show a similar pattern of male-biased European ancestry coupled with female-biased Native American ancestry. This pattern

Table 1. Average ancestral composition of Ecuadorian ethnic groups compared to other admixed American populations

Population	African %	European %	Native American %	East Asian %	Admixture entropy
Afro-Ecuadorian[a]	49.53 (3.05)	13.85 (1.03)	35.86 (2.78)	0.76 (0.05)	1.48
Mestizo[a]	2.39 (0.36)	29.97 (2.12)	66.11 (2.29)	1.52 (0.017)	1.14
Montubio[a]	9.89 (0.66)	38.08 (1.16)	51.36 (1.04)	0.67 (0.11)	1.4
Tsáchila[a]	1.88 (0.40)	10.66 (1.78)	87.12 (2.14)	0.35 (0.07)	0.65
African American (ASW)	76.25 (2.04)	19.52 (1.23)	3.17 (1.25)	1.06 (0.23)	0.99
African Caribbean (ACB)	88.62 (0.81)	10.92 (0.76)	0.07 (0.03)	0.39 (0.19)	0.54
Colombia (CLM)	9.01 (0.82)	64.75 (1.38)	25.22 (0.98)	1.03 (0.07)	1.29
Mexico (MXL)	4.8 (0.29)	46.23 (2.35)	44.52 (2.30)	4.45 (0.23)	1.44
Peru (PEL)	3.01 (0.65)	20.36 (1.34)	73.76 (1.66)	2.87 (0.48)	1.09
Puerto Rico (PUR)	15.01 (0.96)	71.5 (1.00)	12.82 (0.35)	0.67 (0.05)	1.19

Average ancestral compositions (± standard error) are show as percent African, East Asian, European, and Native American ancestry. Admixture entropy is a measure of the ancestral diversity of the population.
[a]Ecuadorian groups.

is most pronounced in the Mestizo and Montubio populations. The Tsáchila group shows a less-pronounced pattern of sex-biased ancestry, with the three median ΔAdmix values are all very close to zero. However, when the admixed Tsáchila individuals (<90% Native American ancestry) are analyzed separately, they do show a strong pattern of European-biased male ancestry and Native American-biased female ancestry (Figure S4).

Subcontinental genetic ancestry

Ancestry-specific haplotypes were leveraged to perform fine-scale, subcontinental ancestry inference for the Ecuadorian samples, with separate analyses run for each of the three continental ancestry components. To do so, continental ancestry-specific genomes were generated by masking haplotypes that correspond to two of the three continental ancestry components. This process yielded three masked genomes per sample: an African-haplotype-only genome, a European-haplotype-only genome, and a Native American-haplotype-only genome. Each ancestry-specific genome was then compared against corresponding reference populations from Africa, Europe, and the Americas to explore the subcontinental ancestral origins for the Ecuadorian ethnic groups.

African origins

African subcontinental ancestry for the Ecuadorian ethnic groups, along with other admixed American populations, was characterized using a panel of 42 African reference populations, 37 of which were sampled from six of the seven main western African regions involved in the transatlantic slave trade.[46–49] We divided these colonial-era African regions into three broad regions based on geographic and genetic affinity of the reference populations: West Africa, West Central Africa, and Southwest Africa (Figure S5). West Africa includes reference populations sampled from Gambia, Sierra Leone, and the Ivory Coast, corresponding to the colonial era slave trading regions of Senegambia, Sierra Leone, and the Windward Coast. West Central Africa includes reference populations sampled from Benin and Nigeria, corresponding to the Bight of Benin. Southwest Africa includes reference populations sampled from Cameroon, Gabon, and Angola, corresponding to the Bight of Biafra and the Loango Coast. We also included East African and Rainforest Hunter Gather (RFHG) African populations for comparison.

The African reference populations from these regions show distinct patterns of ancestry, with coherent patterns of ancestry seen for West African and West Central African populations and diverse ancestry seen for the Bantu-speaking populations of Southwest Africa (Figure 4A; Figure S5). The observed genetic population structure closely mirrors the geographic distribution of the African reference populations, with the cosmopolitan Yaounde population showing admixture between the West Central African and nearby Southwest African groups. The East African and Rainforest Hunter Gatherer populations show distinct patterns of genetic ancestry. The Banbongo population from Gabon shows a mix of Bantu and Rainforest Hunter Gatherer ancestry.

The Ecuadorian groups show varied patterns of African ancestry, with affinities to different admixed American populations (Figure 4A; Table 2). The Afro-Ecuadorians show primarily West Central African ancestry, with similarity to populations from modern-day Benin and Nigeria, followed by West African ancestry, with similarity to populations from Gambia, the Ivory Coast, and Sierra Leone. This pattern of African ancestry is closest to the patterns seen for the African American reference populations and populations from English-speaking countries in the Caribbean.[49] The Montubio and Tsáchila show primarily West African ancestry followed by a West Central African component, which is most similar to populations from Mexico, Central America, Colombia, and Venezuela along

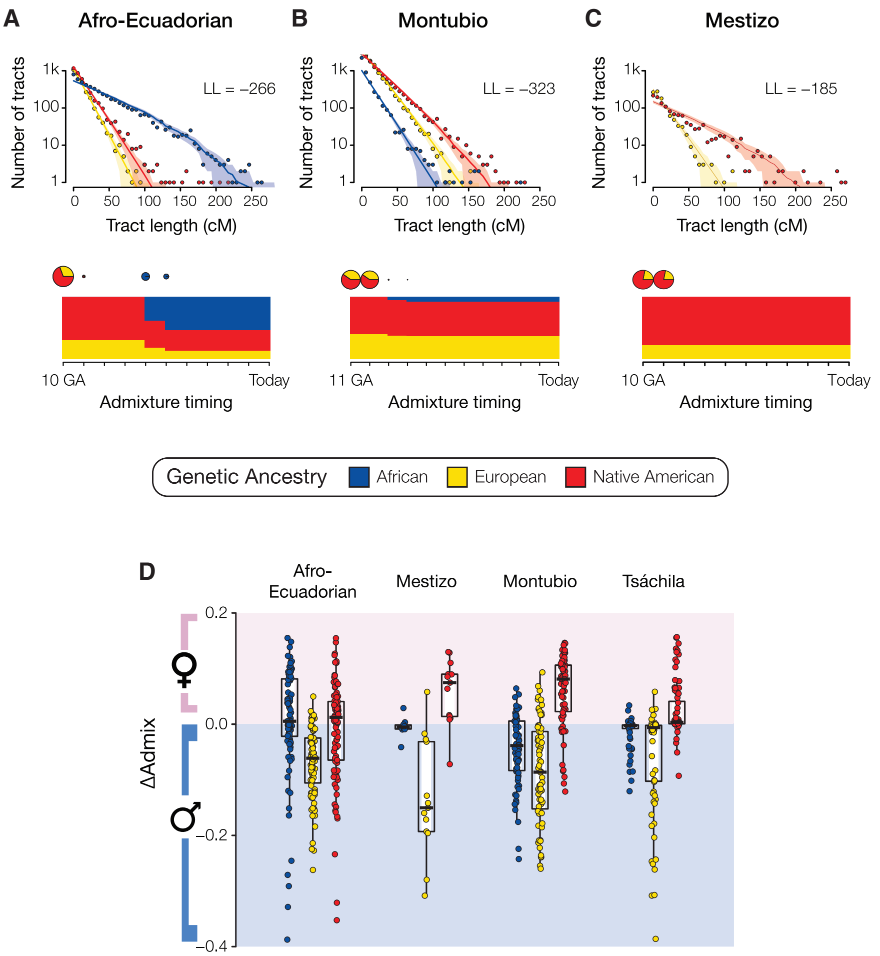

Figure 3. Timing and sex-biased admixture for Ecuadorian ethnic groups
Estimated admixture timings and sex-biased ancestry proportions are shown for each of the three ancestral groups: African (blue), European (yellow), and Native American (red). Admixture timing estimates are shown for (A) Afro-Ecuadorians, (B) Montubios, and (C) Mestizos. On the top panels, the observed (points) and predicted (solid line) ancestry tract size distributions are shown with shaded 95% confidence intervals. LL indicates the log-likelihood values for the models. On the bottom panels, admixture event timings are shown together with ancestry proportions. Each inferred admixture event is indicated by a circle, which is scaled according to the size of the contribution to the population and also shows the relative ancestry proportions. The y-axes of the charts show the inferred continental ancestry fractions, and the x-axes show time as the number of generations ago (GA).
(D) Distributions of ΔAdmix values, which measure differences in ancestry between the X chromosome and the autosomes, are shown for all sampled individuals and each continental ancestry among the four ethnic groups. Values above zero indicate female-biased admixture, and values below zero indicate male-biased admixture.

with Spanish-speaking countries in the Caribbean. The Mestizo population shows the most distinct pattern of African ancestry, with more Southwestern and Bantu ancestry, but this may be an artifact of the small amount of African ancestry seen for this population; there were only 8 Mestizos with enough African ancestry (>3%) to allow for subcontinental ancestry analysis.

We validated our approach to African subcontinental ancestry inference using simulation of admixed genomes containing different combinations of ancestry for the four main African regions analyzed here. Simulated genomes were generated for a range of ancestry fractions (20%–80%) for each of the four African regions, and our subcontinental ancestry inference approach was applied to the simulated admixed genomes. Ancestry inferences for all four of the main African regions show high levels of accuracy when region-specific simulated (expected) ancestry values are compared to observed values generated via the subcontinental ancestry inference approach used here ($R^2 = 0.99$; Figure S6).

European origins

All four Ecuadorian populations show a pattern of European ancestry that is mostly consistent with Spanish ancestry, similar to what is seen for other modern Latin American populations from Colombia, Mexico, Peru, and Puerto Rico (Figure 4C; Table 2). While this is very much unsurprising, it does serve as a positive control for our approach to subcontinental ancestry analysis. The Afro-Ecuadorian population has the highest level of Northern and Central European ancestry, albeit as a minor fraction, which may reflect immigration of laborers from Jamaica starting in the late 19th century.[50] Admixture simulations were used to validate the ability of our subcontinental ancestry inference method to distinguish North and Central European ancestry from South European ancestry. Simulated (expected) and observed European subcontinental ancestry fractions show high correspondence in support our approach ($R^2 = 0.96$).

Native American origins

Given the complex demographic history of Indigenous populations in the Americas, and the high levels of population structure seen for Native American ancestry reference populations, we were not able to directly quantify Native American admixture proportions for the Ecuadorian populations in the same way that was done for their African and European ancestry components. Native American ancestry in the Ecuadorian populations was analyzed via ADMIXTURE, to get a qualitative view of their ancestry

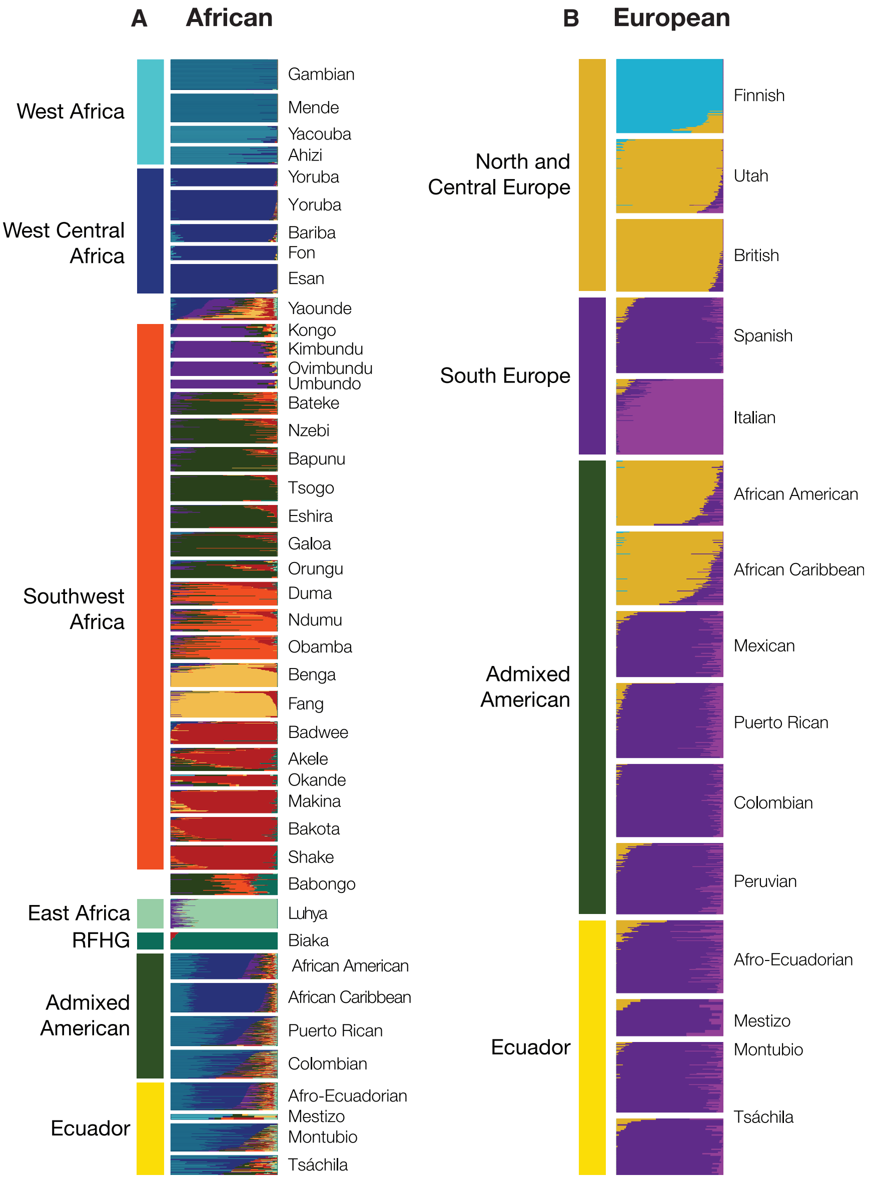

Figure 4. African and European subcontinental ancestry origins for Ecuadorian ethnic groups
Subcontinental ancestral origins are shown separately for (A) African and (B) European continental ancestry components. For each panel, Ecuadorian populations and other modern admixed American populations are compared to a specific panel of continental reference populations. Population groups are indicated to the left of each panel, and individual population labels are shown on the right. Each bar in the plot represents an individual sample, and individuals' subcontinental ancestry components, based on the results of our NNLS analysis, are color-coded according to their group origins. RFHG, Rainforest Hunter Gatherer.

composition, and with phylogenetic analysis to infer the most closely related reference populations. The Native American origins of the Ecuadorian ethnic groups were characterized using a panel of 23 reference populations from Mesoamerican, Central American, Colombian, Amazonian, and Andean tribes (Figure S7), in comparison with modern admixed populations from Colombia, Peru, and Puerto Rico.

ADMIXTURE showed an optimal number of K = 10 ancestry components (Figure S8), and the four Ecuadorian groups form a single, closely related cluster, to the exclusion of all the other admixed American populations and all of the Native American reference populations (Figure 5A). One of the Native American ancestry components (dark red) for the Ecuadorian groups is most pronounced in the Tsáchila and shared mostly, albeit to a small extent, with Indigenous and modern Colombian populations. The minor Native American components for the Ecuador populations (green, yellow, and purple) are also most closely related to nearby Colombian populations and appear to correspond to Andean Indigenous ancestry. Interestingly, the primary Native American ancestry component of the Tsáchila (light red) is substantially less abundant in other Ecuadorian groups and largely absent from the Native American reference populations used here. Thus, similar to what was seen at the continental level, the Tsáchila show two distinct Native American ancestry components, with the less-abundant pattern far more similar to what is seen for the other Ecuadorian groups. The primary Native American component for the Tsáchila may represents an Indigenous source population for Ecuador, for which we do not currently have a reference population, or it could reflect high levels of genetic drift and resulting structure for this population. Phylogenetic analysis and the outgroup f_3 statistic confirm that the Native American component of the Ecuadorian groups is most closely related to Andean Indigenous populations followed by Colombian Indigenous populations (Figure 5B; Figure S9).

Discussion

A major aim of this study was to consider Ecuadorian ethnic identity in the context of the genetic ancestry and origins of the people that make up the country's officially recognized ethnic groups. An emphasis was placed on previously understudied and historically marginalized groups, including Afro-Ecuadorian, Montubio, and Tsáchila

Table 2. Average African and European subcontinental ancestral composition of Ecuadorian ethnic groups

Population	West Africa	West Central Africa	Southwest Africa	East Africa	North and Central Europe	South Europe
Afro-Ecuadorians	25.38 (1.69)	44.92 (1.63)	26.57 (0.86)	2.91 (0.31)	5.59 (1.15)	94.41 (1.15)
Mestizo	52.24 (7.81)	1.87 (1.87)	31.12 (13.79)	14.77 (9.05)	3.09 (1.75)	96.91 (1.75)
Montubio	38.83 (2.65)	20.62 (1.85)	37.21 (3.58)	2.99 (0.42))	0.76 (0.30)	99.24 (0.30)
Tsáchila	40.91 (5.18)	25.97 (4.01)	27.54 (3.46)	4.11 (1.39)	1.46 (0.97)	98.54 (0.97)

Average subcontinental ancestral compositions are show as percentages (± standard error).

populations. The genetic ancestry of the majority Mestizo ethnic group was considered in light of historical knowledge on Ecuadorian population dynamics and the cultural forces related to *mestizaje* and assimilation.

High Native American ancestry in Afro-Ecuadorians

Contrary to our expectations, Afro-Ecuadorians did not show a large majority of African ancestry, although it was the single largest ancestry component on average (49.5%; Table 1), and they did show a high overall level of Native American ancestry (35.9%; Table 1). Afro-Ecuadorians show substantially less African ancestry than seen for the African American and African Caribbean reference populations characterized here, and they have the highest level of admixture seen for any of the four Ecuadorian ethnic groups (admixture entropy = 1.48; Table 1). Even more strikingly, this population has a number of individuals who show very high levels of Native American ancestry with little or no African ancestry (Figures 1 and 2). To our knowledge, this has not been observed for any other Afro-descendant population in the Americas.[21–24,51–54] The high levels of Native American ancestry seen for Afro-Ecuadorians may reflect the historical legacy of the autonomous Afro-descendant communities established in Esmeraldas starting in the 16th century, which included both escaped slaves and members of local Indigenous communities.[10,11] This finding underscores the extent to which ethnic identity in Ecuador can serve as a marker of shared culture rather than common ancestry.

Social construction of Mestizos in Ecuador

The Mestizo ethnic group shows an average of 66.1% Native American ancestry, which is higher than any other admixed American population studied here except for Peru. As with the Afro-Ecuadorians, there are a number of individuals who identify as Mestizo but have almost entirely Native American ancestry (Figures 1 and 2). After Bolivia (62%), Peru (24%) has the second-largest Indigenous population in South America, whereas the Indigenous Amerindian group in Ecuador makes up only 7% of the population. Thus, one may not expect to see such high levels of Native American ancestry in Ecuador's majority Mestizo population. The pattern we observe could be explained by historical records and sociological studies of Ecuador that indicate a relatively low founding immigrant population from Spain coupled with cultural forces that led many Indigenous people to adopt a Mestizo identity.[8,18,55] In much of Ecuador, Mestizo came to imply someone who was fluent in Spanish and who lived in and around urban centers, irrespective of their ancestry. Indigenous people who migrated to cities and learned Spanish would either be ascribed, or adopt, a Mestizo identity, and in so doing gain access to a broader, shared national identity. This cultural assimilation had the effect of marginalizing Indigenous identity and communities, while also leaving out Afro-Ecuadorians whose markers of ancestry were harder to ignore. Our genetic ancestry results are consistent with the social construction of a Mestizo identity in Ecuador that is distinct from ancestrally grounded Mestizo identities in other Latin American countries.

Montubio ancestry and admixture

The complex relationship between ethnic identity and ancestry in Ecuador is exemplified by the newly recognized Montubio ethnic group. Montubios live primarily in the coastal region of Ecuador—in the provinces of Manabí, Guayas, Los Rios, and El Oro—and their cultural identity is tied to a distinctly rural and agrarian lifestyle. Given their recent origins and recognition, this group stands out as a counterexample to the narrative that ethnic identities in Latin America were formed in the early colonial period, via the process of *mestizaje*, and have remained largely unchanged over centuries. Some scholars have claimed that Montubios are essentially another Mestizo group, with respect to mixed Spanish and Native American ancestry, albeit with a distinct cultural heritage.[7] However, other scholars have pointed to African contributions to Montubio ancestry and culture, consistent with their rural location in the province of Manabí, which also has a large Afro-Ecuadorian population.[56]

Our results highlight the admixed nature of the Montubio population. The main ancestry component is Native American, followed closely by European ancestry, with a smaller but not insubstantial African component (Figures 1 and 2). Montubios show the second-highest overall level of admixture seen for any of the admixed American populations studied here (admixture entropy = 1.40; Table 1). The high levels of Native American ancestry in the Montubio are similar to what is seen for all other Ecuadorian ethnic groups and consistent with the relatively low number of European immigrants who contributed to the

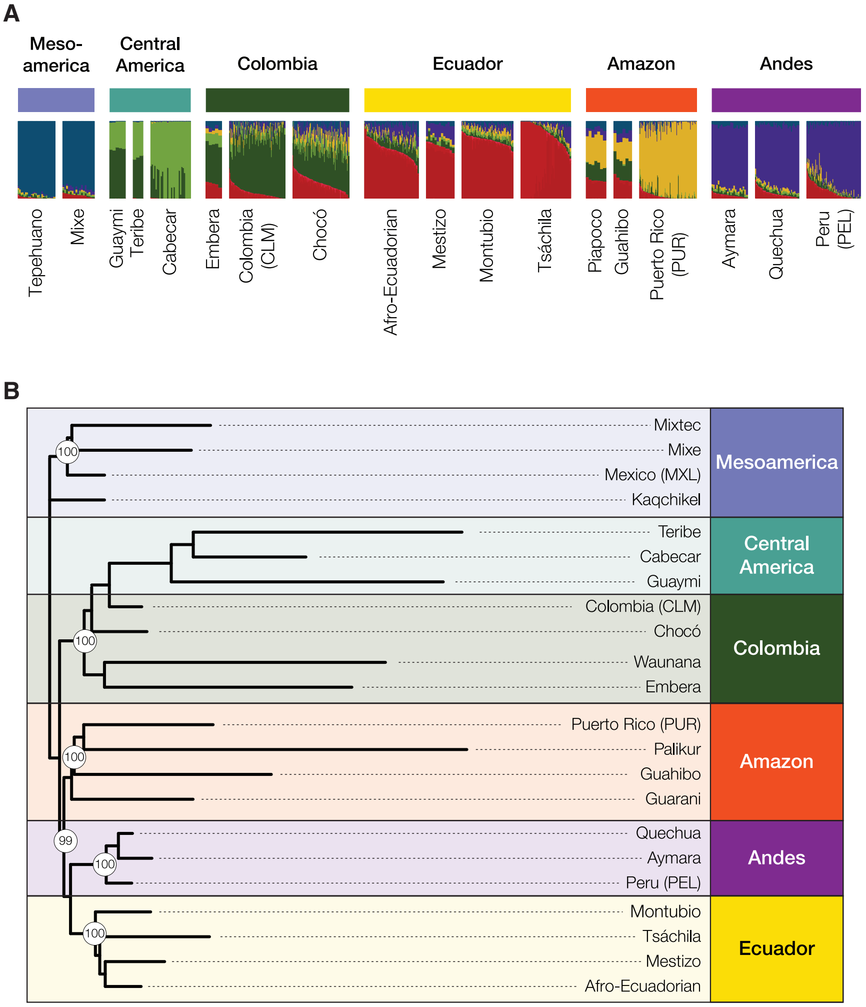

Figure 5. Native American subcontinental ancestry origins for Ecuadorian ethnic groups

(A) Ecuadorian populations and other modern admixed American populations are compared to a specific panel of Native American reference populations. Population groups are indicated to the left of each panel, and individual population labels are shown on the right. Each bar in the plot represents an individual sample, and individuals' subcontinental ancestry components are color-coded according to their group origins.

(B) Phylogenetic tree showing the relationship between the Native American ancestry component of Ecuadorian populations, other admixed American populations, and Native American reference populations.

modern population. The minor but still notable African ancestry component supports historical and sociological studies that emphasize African cultural contributions to the Montubio people.

Two distinct ancestry groups in the Tsáchila

Overall, the Tsáchila show a very high level of Native American ancestry (87.1%; Table 1). However, closer inspection of this ethnic group shows clear evidence for two distinct ancestry groups. At the continental level, roughly two-thirds of individuals show almost entirely Native American ancestry, with the remaining individuals showing various levels of primarily European admixture (Figures 1 and 2). This same grouping can be seen for the Native American subcontinental ancestry of the Tsáchila (Figure 5A). There is a primary Native American ancestry component that is entirely unique to the Tsáchila (light red) and a secondary component that much more closely resembles the Native American ancestry of the Ecuadorian Mestizos (dark red).

The Tsáchila individuals studied here were sampled from the city of Santo Domingo de los Colorados located in the province of Santo Domingo de los Tsáchilas, which, as the name suggests, is the historical homeland of the group. Even the name of the city "de los Colorados" (of the dyed) refers to the Tsáchila ethnic group and their custom of covering themselves in the red juices of achiote seeds to prevent smallpox infection. Thus, it may be the case that residents of Santo Domingo de los Colorados with Spanish and Native American ancestry, who may be expected to ethnically identify as Mestizo, identify as Tsáchila. This would be an interesting example of individuals from a relatively high-status majority group choosing to identify with a historically oppressed Indigenous group. On the other hand, Tsáchila ethnic identity may provide social advantages for Mestizo individuals who reside in their Indigenous homeland. The distinction between ancestry and ethnic identity among the Tsáchila Santo Domingo de los Colorados also suggests the possibility that ethnic identity in Ecuador is strongly influenced by local geographic origins and culture.

Conclusions

The results reported here show how genetic ancestry co-varies with ethnicity in Ecuador: Mestizos and Montubios are primarily admixed with Spanish and Native American ancestry, whereas the Tsáchila and the Afro-Ecuadorians show the highest levels of Native American and African ancestry, respectively. All four ethnic groups show evidence of sex-biased admixture with greater levels of male European and female Native American ancestry, consistent with the historical record. Nonetheless, we find the exceptions to these general trends to be the most interesting and revealing findings. We observed several unexpected patterns of genetic ancestry for different ethnic groups, which underscore the extent to which ethnic identity in Ecuador is shaped by both culture and ancestry. Mestizos show

surprisingly high levels of Native American ancestry, when considered together with the size of the Indigenous population in the country, pointing to the role of language and cultural assimilation in the formation of this ethnic group. The Afro-Ecuadorians show the lowest levels of African ancestry, and the highest levels of Native American ancestry, seen for any Afro-descendant population in the Americas. This population includes a number of individuals with almost entirely Native American ancestry, pointing to the possibility of a distinctly African cultural identity for the region, shaped by its unique history.

Data and code availability

The Ecuador genotype data supporting the current study have not been deposited in a public repository because the data are not public. They data are available from the corresponding author on request.

Supplemental information

Supplemental information can be found online at https://doi.org/10.1016/j.xhgg.2021.100050.

Acknowledgments

S.D.N. was supported by the Georgia Tech Bioinformatics Graduate Program. A.B.C., A.T.C., L.R., and I.K.J. were supported by the IHRC-Georgia Tech Applied Bioinformatics Laboratory. S.S. and L.M.R. were supported by the Division of Intramural Research (DIR) of the National Institute on Minority Health and Health Disparities (NIMHD) at the National Institutes of Health (NIH) , and L.M.R. was supported by the NIH Distinguished Scholars Program (DSP).

Declaration of interests

The authors declare no competing interests.

Received: January 19, 2021
Accepted: August 9, 2021

Web resources

1000 Genomes Genetic Maps, https://github.com/joepickrell/1000-genomes-genetic-maps

References

1. Lauderbaugh, G. (2012). The History of Ecuador (ABC-CLIO).
2. Schwartz, S.B. (1995). Colonial identities and the sociedad de castas. Colonial Lat. Am. Rev. *4*, 185–201.
3. Lewis, L.A. (2012). Between 〉Casta〈 and 〉Raza. In Race and Blood in the Iberian World, D.N.M. Elena Martínez and M.-S. Hering Torres, eds. (Münster, Germany: LIT Verlag), p. 99.
4. INEC (2010). Población y Demografía de Ecuador. https://www.ecuadorencifras.gob.ec/censo-de-poblacion-y-vivienda/.
5. El Universo (2011). Población del país es joven y mestiza, dice censo del INEC. https://www.eluniverso.com/2011/09/02/1/1356/poblacion-pais-joven-mestiza-dice-censo-inec.html.
6. Ochoa, P.P.J. (2019). Huelguistas montuvios. In El Telêgrafo (Guayaquil: Ecuador).
7. Paredes Ramirez, W. (2005). Los Montubios y Nosotros (Corporación Montubia del Litoral).
8. Roitman, K. (2008). Hybridity, Mestizaje, and Montubios in Ecuador. QEH Working Paper Series, Working Paper 165.
9. Mathewson, K. (2008). Coastal Ecuador's Montubios in Ethnogeographic and Historical Perspective. In Ethno- and Historical Geographic Studies in Latin America: Essays in Honor of William V. Davidson, P.H. Herlihy, K. Mathewson, and C.S. Revels, eds. (Baton Rouge: Geoscience Publications), pp. 239–262.
10. José Balda, P.M., García Salazar, J., and Chalá Angulo, C. (2009). Enciclopedia del saber Afroecuatoriano (Quito, Ecuador: Vicariato Apostólico de Esmeraldas; Centro Cultural Afroecuatoriano).
11. Rueda Novoa, A.R. (2010). De esclavizados a comuneros en la cuenca aurífera del río Santiago-río Cayapas (Esmeraldas). Etnicidad negra en construcción en Ecuador Siglos XVIII-XIX. PhD Thesis. (Quito, Ecuador: Universidad Andina Simón Bolívar).
12. de la Cadena, M. (2000). Indigenous Mestizos: the politics of race and culture in Cuzco, Peru, 1919-1991 (Duke University Press).
13. Miller, M.G. (2009). Rise and Fall of the Cosmic Race: The Cult of Mestizaje in Latin America (University of Texas Press).
14. Martínez-Echazábal, L. (1998). Mestizaje and the discourse of national/cultural identity in Latin America, 1845-1959. Lat. Am. Perspect. *25*, 21–42.
15. Ibarra, H. (1998). La otra cultura: imaginarios, mestizaje y modernización (Abya-Yala).
16. Santangelo, R., González-Andrade, F., Børsting, C., Torroni, A., Pereira, V., and Morling, N. (2017). Analysis of ancestry informative markers in three main ethnic groups from Ecuador supports a trihybrid origin of Ecuadorians. Forensic Sci. Int. Genet. *31*, 29–33.
17. Zambrano, A.K., Gaviria, A., Cobos-Navarrete, S., Gruezo, C., Rodríguez-Pollit, C., Armendáriz-Castillo, I., García-Cárdenas, J.M., Guerrero, S., López-Cortés, A., Leone, P.E., et al. (2019). The three-hybrid genetic composition of an Ecuadorian population using AIMs-InDels compared with autosomes, mitochondrial DNA and Y chromosome data. Sci. Rep. *9*, 9247.
18. de la Cadena, M. (2005). Are "Mestizos" Hybrids? The Conceptual Politics of Andean Identities. J. Lat. Am. Stud. *37*, 259–284.
19. Homburger, J.R., Moreno-Estrada, A., Gignoux, C.R., Nelson, D., Sanchez, E., Ortiz-Tello, P., Pons-Estel, B.A., Acevedo-Vasquez, E., Miranda, P., Langefeld, C.D., et al. (2015). Genomic Insights into the Ancestry and Demographic History of South America. PLoS Genet. *11*, e1005602.
20. Agencia Española de Cooperación Internacional para el Desarrollo (2021). Afrodescendientes: Programa de Coopercaión con Afrodescendientes. https://www.aecid.es/ES/d%C3%B3nde-cooperamos/alc/programas-horizontales/programa-afrodescendientes.
21. Fortes-Lima, C., Gessain, A., Ruiz-Linares, A., Bortolini, M.C., Migot-Nabias, F., Bellis, G., Moreno-Mayar, J.V., Restrepo, B.N., Rojas, W., Avendaño-Tamayo, E., et al. (2017). Genome-wide Ancestry and Demographic History of African-Descendant Maroon Communities from French Guiana and Suriname. Am. J. Hum. Genet. *101*, 725–736.
22. Jordan, I.K., Rishishwar, L., and Conley, A.B. (2019). Native American admixture recapitulates population-specific

migration and settlement of the continental United States. PLoS Genet. *15*, e1008225.

23. Baharian, S., Barakatt, M., Gignoux, C.R., Shringarpure, S., Errington, J., Blot, W.J., Bustamante, C.D., Kenny, E.E., Williams, S.M., Aldrich, M.C., and Gravel, S. (2016). The Great Migration and African-American Genomic Diversity. PLoS Genet. *12*, e1006059.
24. Bryc, K., Durand, E.Y., Macpherson, J.M., Reich, D., and Mountain, J.L. (2015). The genetic ancestry of African Americans, Latinos, and European Americans across the United States. Am. J. Hum. Genet. *96*, 37–53.
25. Bien, S.A., Wojcik, G.L., Zubair, N., Gignoux, C.R., Martin, A.R., Kocarnik, J.M., Martin, L.W., Buyske, S., Haessler, J., Walker, R.W., et al.; PAGE Study (2016). Strategies for Enriching Variant Coverage in Candidate Disease Loci on a Multiethnic Genotyping Array. PLoS ONE *11*, e0167758.
26. Auton, A., Brooks, L.D., Durbin, R.M., Garrison, E.P., Kang, H.M., Korbel, J.O., Marchini, J.L., McCarthy, S., McVean, G.A., Abecasis, G.R.; and 1000 Genomes Project Consortium (2015). A global reference for human genetic variation. Nature *526*, 68–74.
27. Chang, C.C., Chow, C.C., Tellier, L.C., Vattikuti, S., Purcell, S.M., and Lee, J.J. (2015). Second-generation PLINK: rising to the challenge of larger and richer datasets. Gigascience *4*, 7.
28. Manichaikul, A., Mychaleckyj, J.C., Rich, S.S., Daly, K., Sale, M., and Chen, W.M. (2010). Robust relationship inference in genome-wide association studies. Bioinformatics *26*, 2867–2873.
29. Delaneau, O., Zagury, J.F., and Marchini, J. (2013). Improved whole-chromosome phasing for disease and population genetic studies. Nat. Methods *10*, 5–6.
30. Patin, E., Lopez, M., Grollemund, R., Verdu, P., Harmant, C., Quach, H., Laval, G., Perry, G.H., Barreiro, L.B., Froment, A., et al. (2017). Dispersals and genetic adaptation of Bantu-speaking populations in Africa and North America. Science *356*, 543–546.
31. Reich, D., Patterson, N., Campbell, D., Tandon, A., Mazieres, S., Ray, N., Parra, M.V., Rojas, W., Duque, C., Mesa, N., et al. (2012). Reconstructing Native American population history. Nature *488*, 370–374.
32. Wickham, H. (2016). ggplot2: elegant graphics for data analysis (Springer).
33. R Core Team (2013). R: A language and environment for statistical computing.
34. Alexander, D.H., Novembre, J., and Lange, K. (2009). Fast model-based estimation of ancestry in unrelated individuals. Genome Res. *19*, 1655–1664.
35. Maples, B.K., Gravel, S., Kenny, E.E., and Bustamante, C.D. (2013). RFMix: a discriminative modeling approach for rapid and robust local-ancestry inference. Am. J. Hum. Genet. *93*, 278–288.
36. Lawson, D.J., Hellenthal, G., Myers, S., and Falush, D. (2012). Inference of population structure using dense haplotype data. PLoS Genet. *8*, e1002453.
37. Author (2012). The Lawson-Hanson algorithm for non-negative least squares (NNLS) (Vienna, Austria: R Foundation for Statistical Computing).
38. Moreno-Estrada, A., Gignoux, C.R., Fernández-López, J.C., Zakharia, F., Sikora, M., Contreras, A.V., Acuña-Alonzo, V., Sandoval, K., Eng, C., Romero-Hidalgo, S., et al. (2014). Human genetics. The genetics of Mexico recapitulates Native American substructure and affects biomedical traits. Science *344*, 1280–1285.
39. Patterson, N., Price, A.L., and Reich, D. (2006). Population structure and eigenanalysis. PLoS Genet. *2*, e190.
40. Saitou, N., and Nei, M. (1987). The neighbor-joining method: a new method for reconstructing phylogenetic trees. Mol. Biol. Evol. *4*, 406–425.
41. Tamura, K., Stecher, G., Peterson, D., Filipski, A., and Kumar, S. (2013). MEGA6: Molecular Evolutionary Genetics Analysis version 6.0. Mol. Biol. Evol. *30*, 2725–2729.
42. Paradis, E., Claude, J., and Strimmer, K. (2004). APE: Analyses of Phylogenetics and Evolution in R language. Bioinformatics *20*, 289–290.
43. Gravel, S. (2012). Population genetics models of local ancestry. Genetics *191*, 607–619.
44. Gravel, S., Zakharia, F., Moreno-Estrada, A., Byrnes, J.K., Muzzio, M., Rodriguez-Flores, J.L., Kenny, E.E., Gignoux, C.R., Maples, B.K., Guiblet, W., et al.; 1000 Genomes Project (2013). Reconstructing Native American migrations from whole-genome and whole-exome data. PLoS Genet. *9*, e1004023.
45. Rishishwar, L., Conley, A.B., Wigington, C.H., Wang, L., Valderrama-Aguirre, A., and Jordan, I.K. (2015). Ancestry, admixture and fitness in Colombian genomes. Sci. Rep. *5*, 12376.
46. Eltis, D., and Richardson, D. (2010). Atlas of the Transatlantic Slave Trade. African Diaspora Archaeology Newsletter *13*, 17.
47. Fortes-Lima, C., and Dugoujon, J.-M. (2018). Revisiting genetic ancestry in African Diaspora communities from Atlantic South America. In Trends in Biological Anthropology, M. Holst and M. Alexander, eds. (Oxford, England: Oxbow Books), pp. 9–17.
48. Klein, H.S. (2010). The Atlantic Slave Trade (Cambridge University Press).
49. Micheletti, S.J., Bryc, K., Ancona Esselmann, S.G., Freyman, W.A., Moreno, M.E., Poznik, G.D., Shastri, A.J., Beleza, S., Mountain, J.L.; and 23andMe Research Team (2020). Genetic Consequences of the Transatlantic Slave Trade in the Americas. Am. J. Hum. Genet. *107*, 265–277.
50. Ramsay, P. (2015). Early Jamaican migration to Ecuador and influence. https://jamaica-gleaner.com/article/news/20150821/early-jamaican-migration-ecuador-and-influence.
51. Conley, A.B., Rishishwar, L., Norris, E.T., Valderrama-Aguirre, A., Mariño-Ramírez, L., Medina-Rivas, M.A., and Jordan, I.K. (2017). A Comparative Analysis of Genetic Ancestry and Admixture in the Colombian Populations of Chocó and Medellín. G3 (Bethesda) *7*, 3435–3447.
52. Mathias, R.A., Taub, M.A., Gignoux, C.R., Fu, W., Musharoff, S., O'Connor, T.D., Vergara, C., Torgerson, D.G., Pino-Yanes, M., Shringarpure, S.S., et al.; CAAPA (2016). A continuum of admixture in the Western Hemisphere revealed by the African Diaspora genome. Nat. Commun. *7*, 12522.
53. Tishkoff, S.A., Reed, F.A., Friedlaender, F.R., Ehret, C., Ranciaro, A., Froment, A., Hirbo, J.B., Awomoyi, A.A., Bodo, J.M., Doumbo, O., et al. (2009). The genetic structure and history of Africans and African Americans. Science *324*, 1035–1044.
54. Zakharia, F., Basu, A., Absher, D., Assimes, T.L., Go, A.S., Hlatky, M.A., Iribarren, C., Knowles, J.W., Li, J., Narasimhan, B., et al. (2009). Characterizing the admixed African ancestry of African Americans. Genome Biol. *10*, R141.
55. Clark, A. (1998). Race,'culture,'and mestizaje: the statistical construction of the Ecuadorian Nation, 1930–1950. Journal of Historical Sociology *11*, 185–211.
56. Murra, J. (1963). The Historic Tribes of Ecudor. In Handbook of South American Indians, J.H. Steward, ed. (New York: Cooper Square Publishers), pp. 785–821.

HGGA, Volume *2*

Supplemental information

Genetic ancestry and ethnic identity in Ecuador

Shashwat Deepali Nagar, Andrew B. Conley, Aroon T. Chande, Lavanya Rishishwar, Shivam Sharma, Leonardo Mariño-Ramírez, Gabriela Aguinaga-Romero, Fabricio González-Andrade, and I. King Jordan

Supplementary material

Genetic ancestry and ethnic identity in Ecuador

Shashwat D. Nagar, Andrew B. Conley, Aroon T. Chande, Lavanya Rishishwar, Shivam Sharma, Leonardo Mariño-Ramírez, Gabriela Aguinaga-Romero, Fabricio González-Andrade, and I. King Jordan

Figure S1. Genotype data harmonization and analysis workflow. 2

Figure S2. Principal component analysis (PCA) of the genomic relationship matrix for the four Ecuadorian populations compared to reference populations from Africa, the Americas, East Asia, and Europe. 3

Figure S3. ADMIXTURE plots showing continental ancestry fractions for individuals from (A) African, Admixed American, East Asian, and European reference populations and (B) the four Ecuadorian ethnic groups. 4

Figure S4. Sex-biased admixture for Tsáchila subgroups. 5

Figure S5. African reference populations used for this study. 6

Figure S6. Validation of African subcontinental ancestry inference. 7

Figure S7. Native American reference populations used for this study. 8

Figure S8. Cross-validation error values (y-axis) for AMDIXTURE run over a range of ancestry component values K=2-12 on Native American ancestry.. 9

Figure S9. Native American origins of Ecuadorian populations. 10

Table S1. Reference populations used in this study. 11

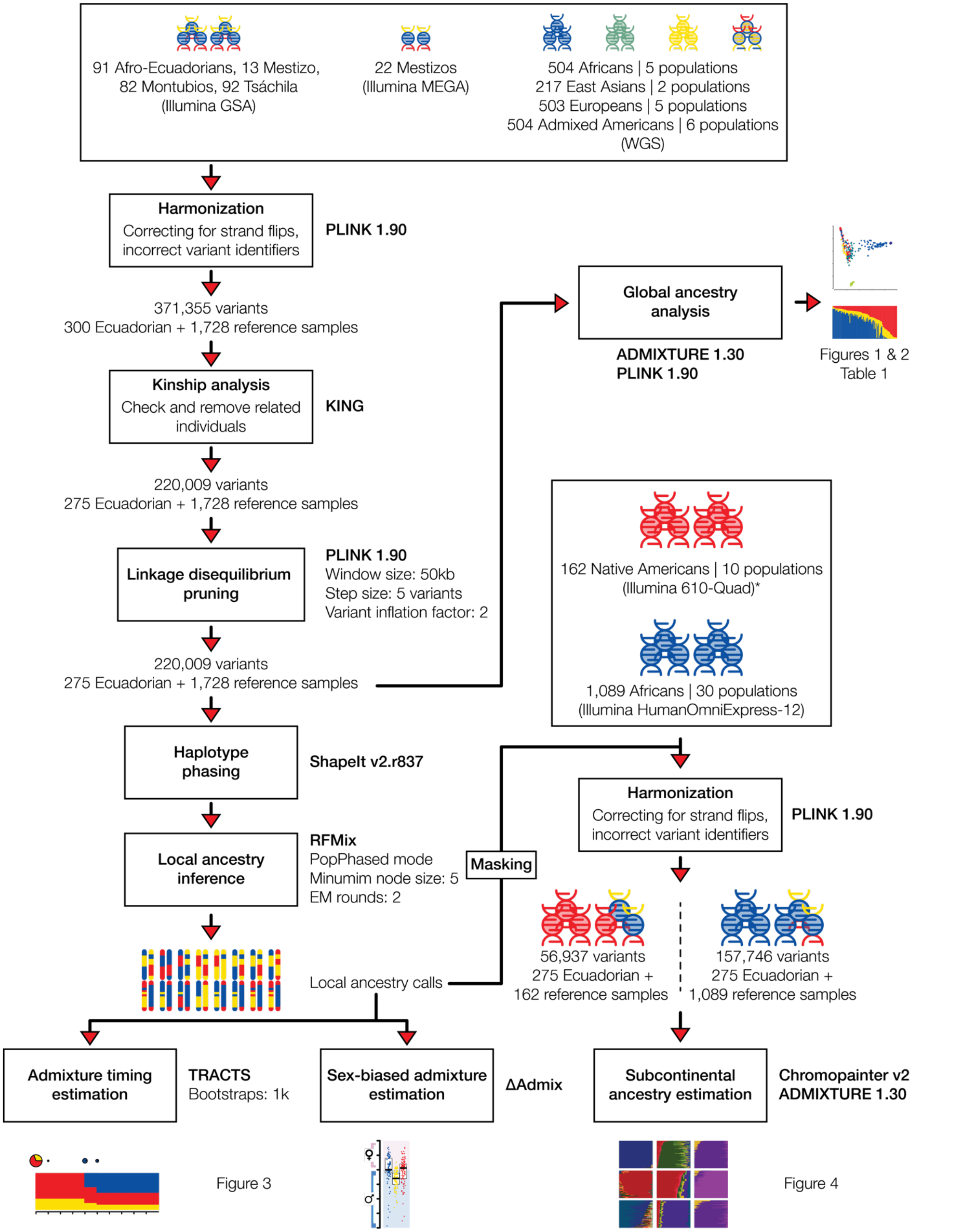

Figure S1. **Genotype data harmonization and analysis workflow.** Different datasets, harmonization and analysis steps, along with tools used at each step are illustrated in this workflow.

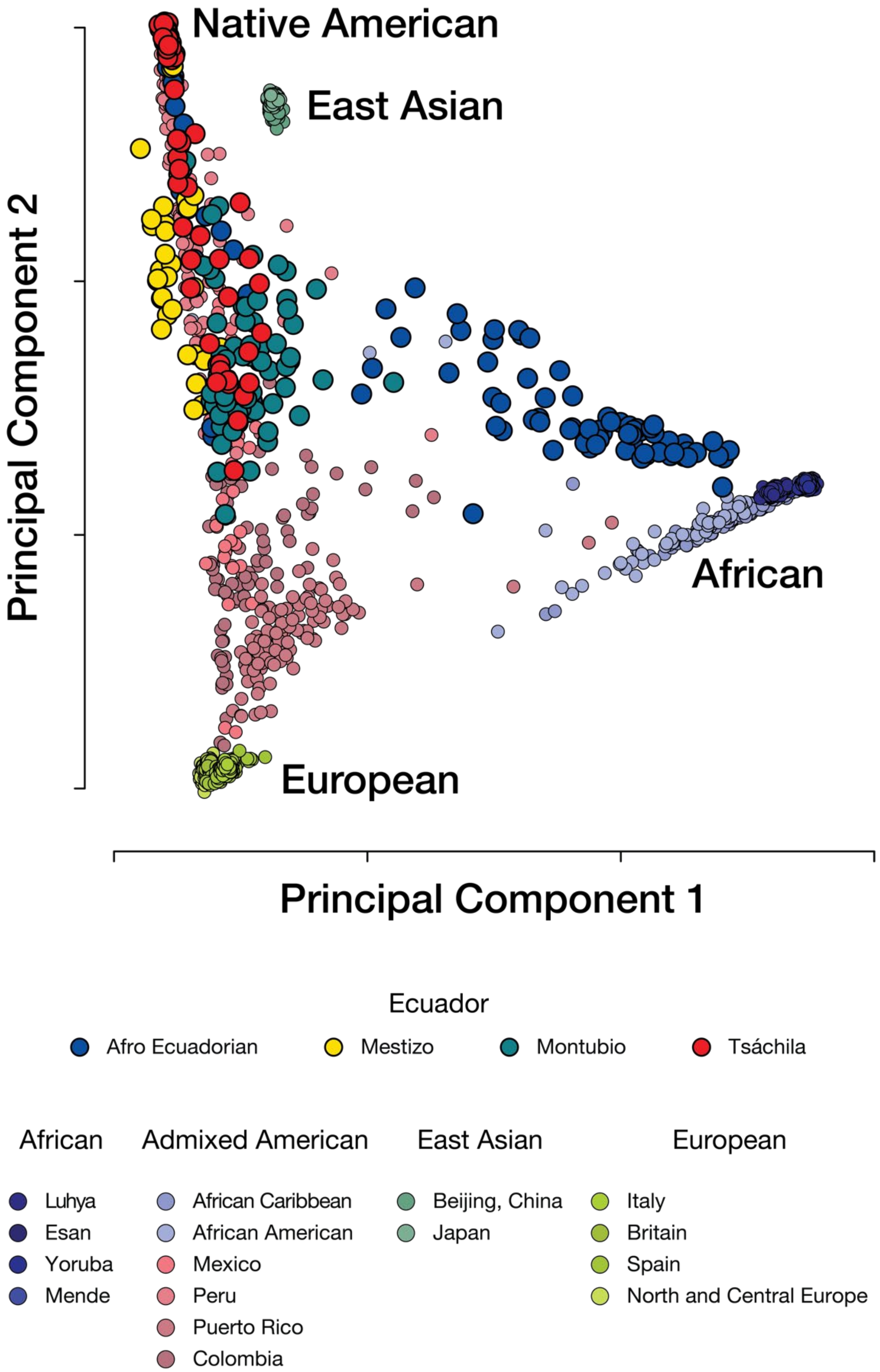

Figure S2. **Principal component analysis (PCA) of the genomic relationship matrix for the four Ecuadorian populations compared to reference populations from Africa, the Americas, East Asia, and Europe.**

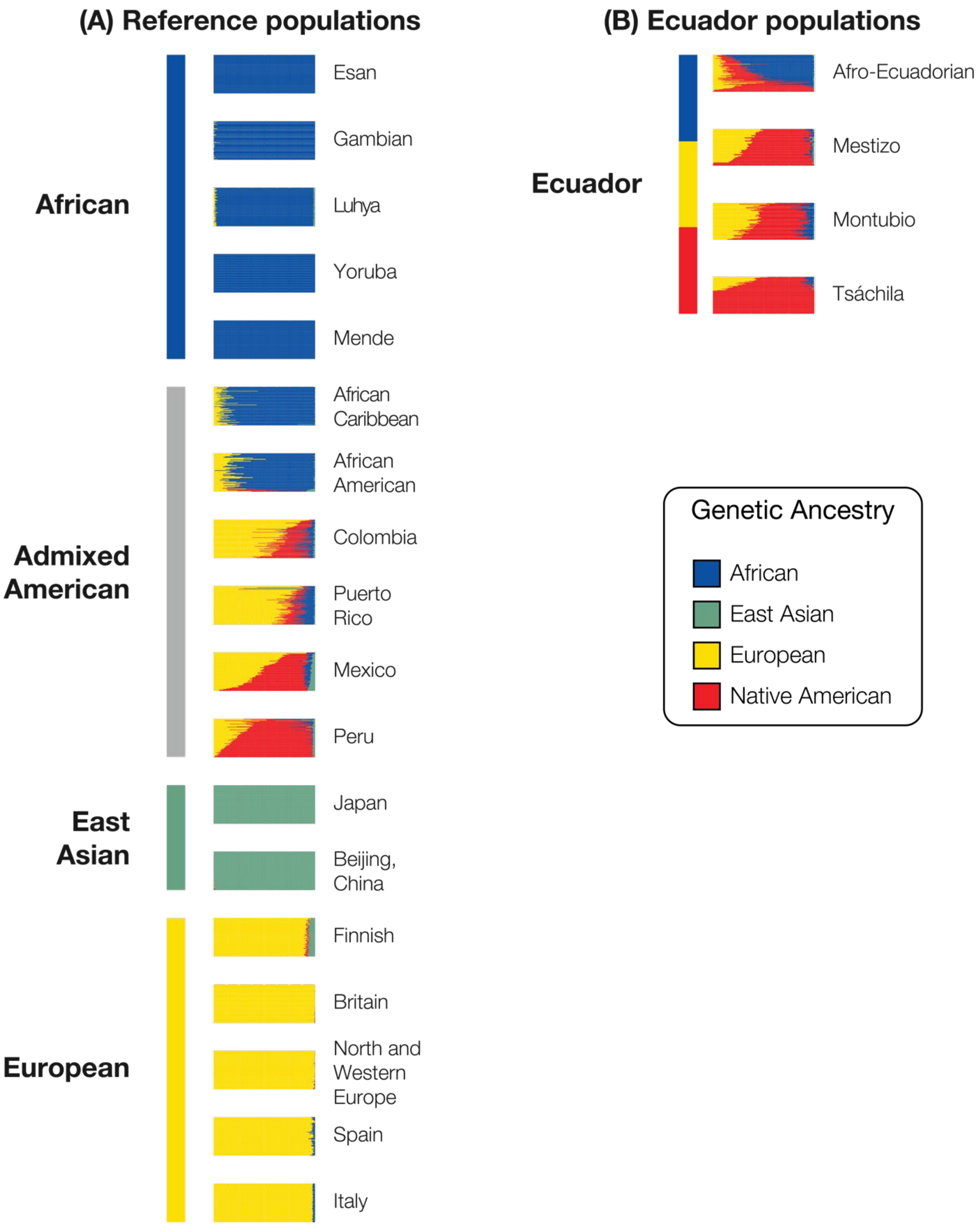

Figure S3. **ADMIXTURE plots showing continental ancestry fractions for individuals from (A) African, Admixed American, East Asian, and European reference populations and (B) the four Ecuadorian ethnic groups.**

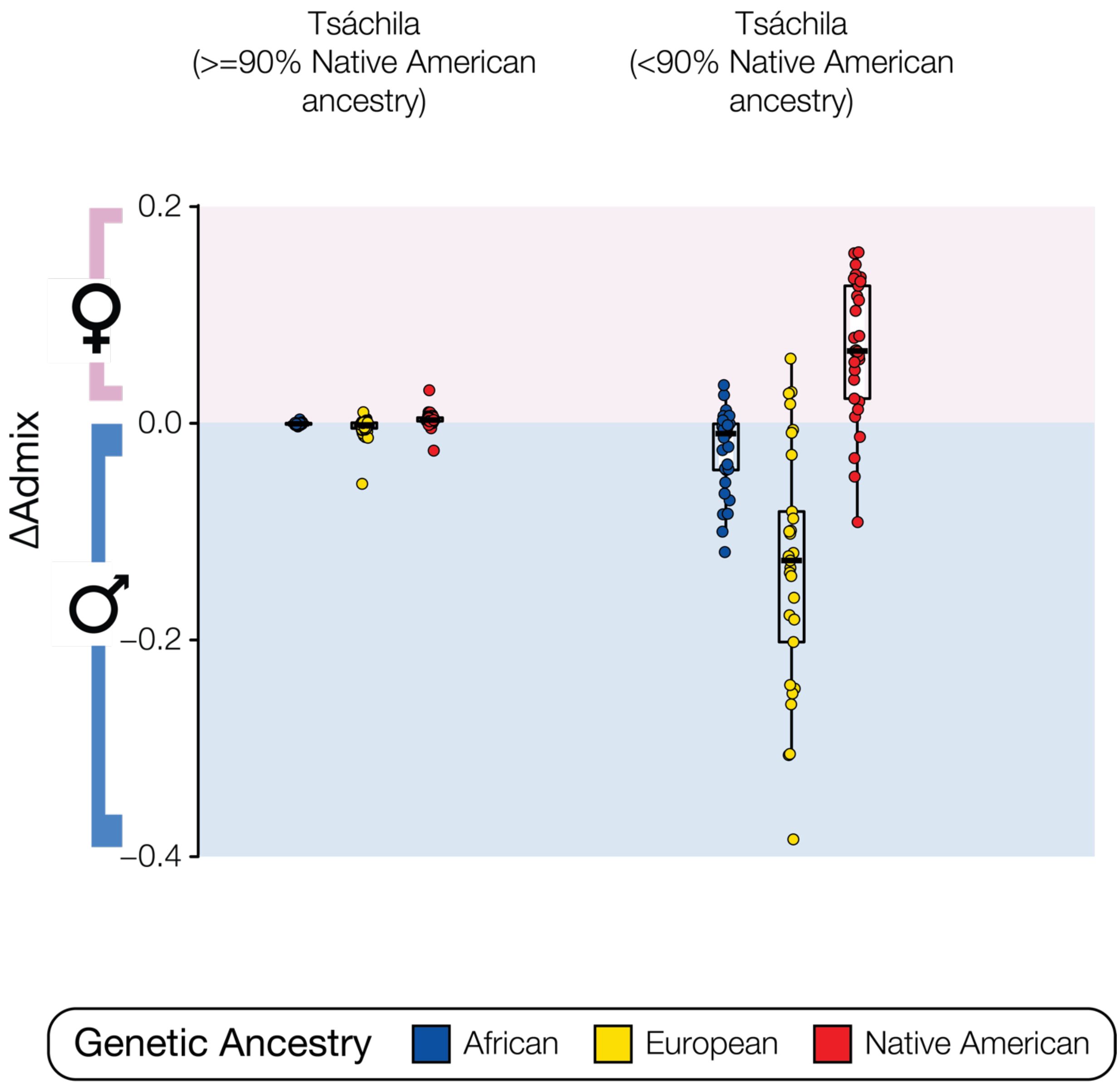

Figure S4. **Sex-biased admixture for Tsáchila subgroups.** Sex-biased ancestry proportions are shown for each of the three ancestral groups: African (blue), European (yellow), and Native American (red). Results are shown for (A) non-admixed individuals (>=90% Native American ancestry) and (B) admixed individuals (<90% Native American ancestry).

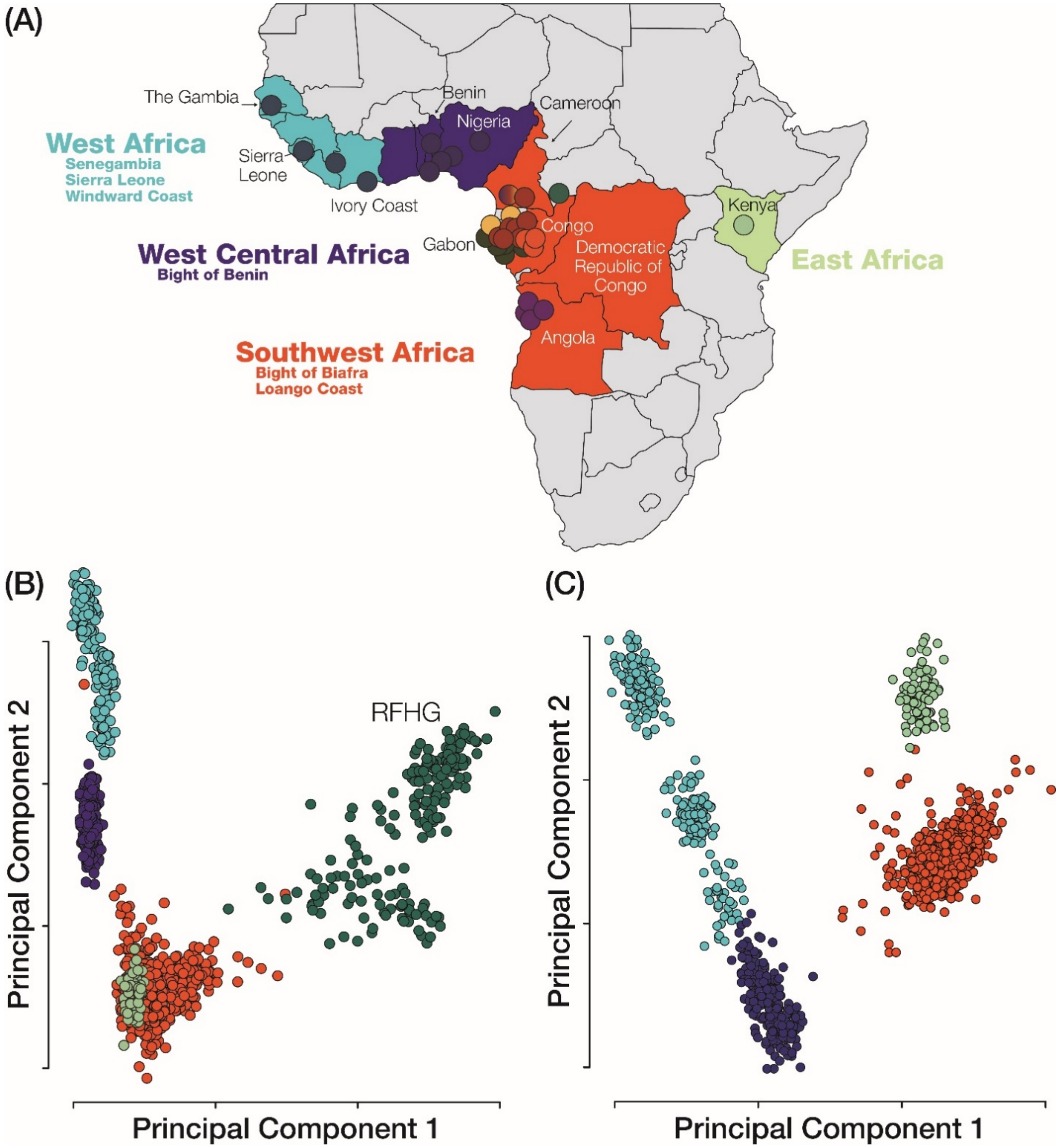

Figure S5. **African reference populations used for this study.** (A) The sampling locations of reference populations are shown, with respect to modern African countries, along with the locations of the four main geographic regions – West Africa, West Central Africa, Southwest Africa, and East Africa – and the corresponding colonial era slave trading regions along the west coast of Africa. (B&C) Principal components analyses (PCA) showing the genetic relationships among African reference population samples from the four geographic regions. Panel B includes rainforest hunter gatherer (RFHG) populations, and panel C includes only populations from the four main African regions analyzed here.

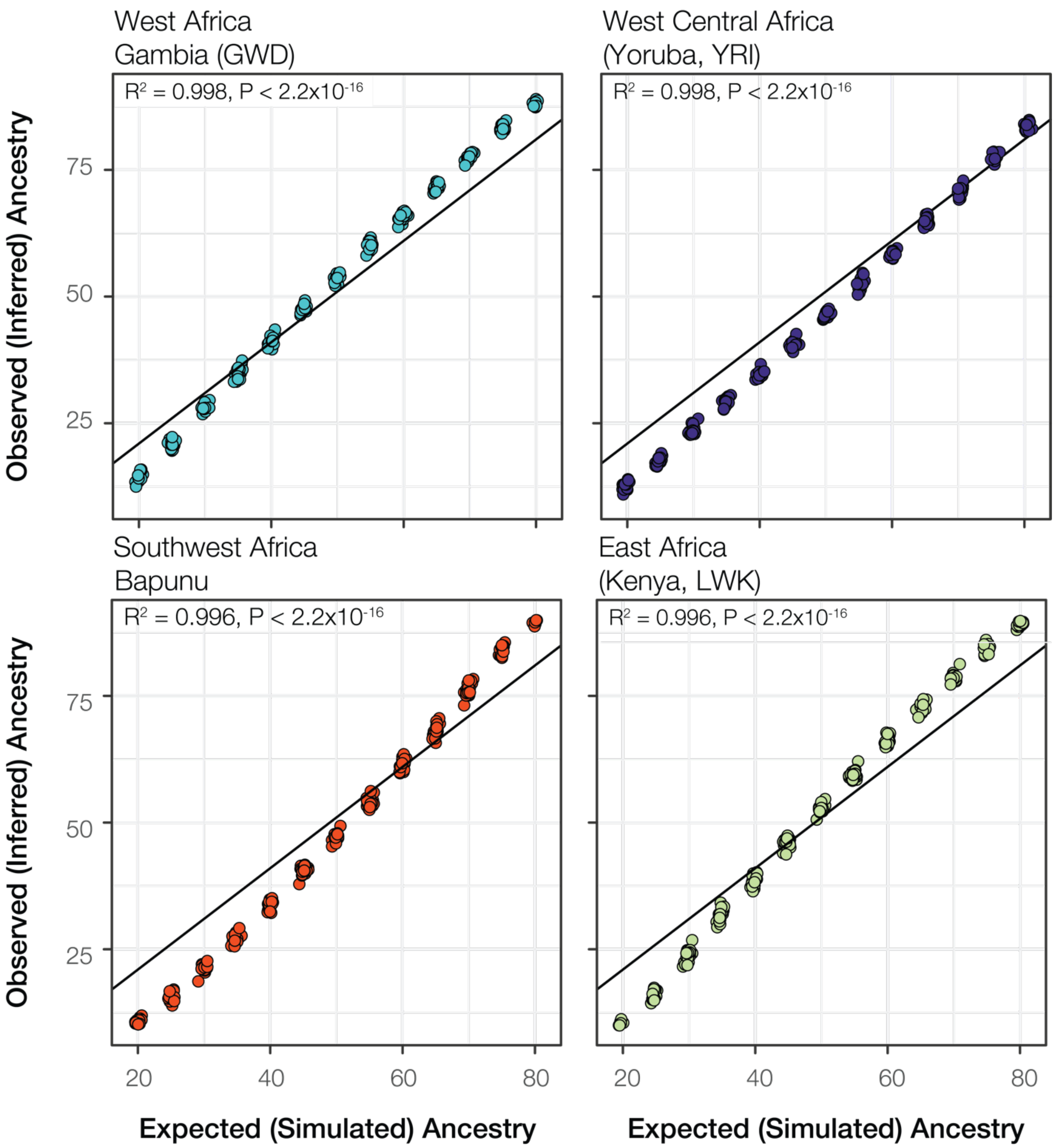

Figure S6. **Validation of African subcontinental ancestry inference.** Expected levels of ancestry based on simulated admixed genomes (x-axis) are compared to observed levels of ancestry based on NNLS method applied to simulated genomes (y-axis). Results are shown for the four main geographic regions studied here: West Africa (Gambia, GWD), West Central Africa (Yoruba, YRI), Southwest Africa (Bapunu), and East Africa (Kenya, LWK). Pearson correlation R2 and P-values are shown for each comparison.

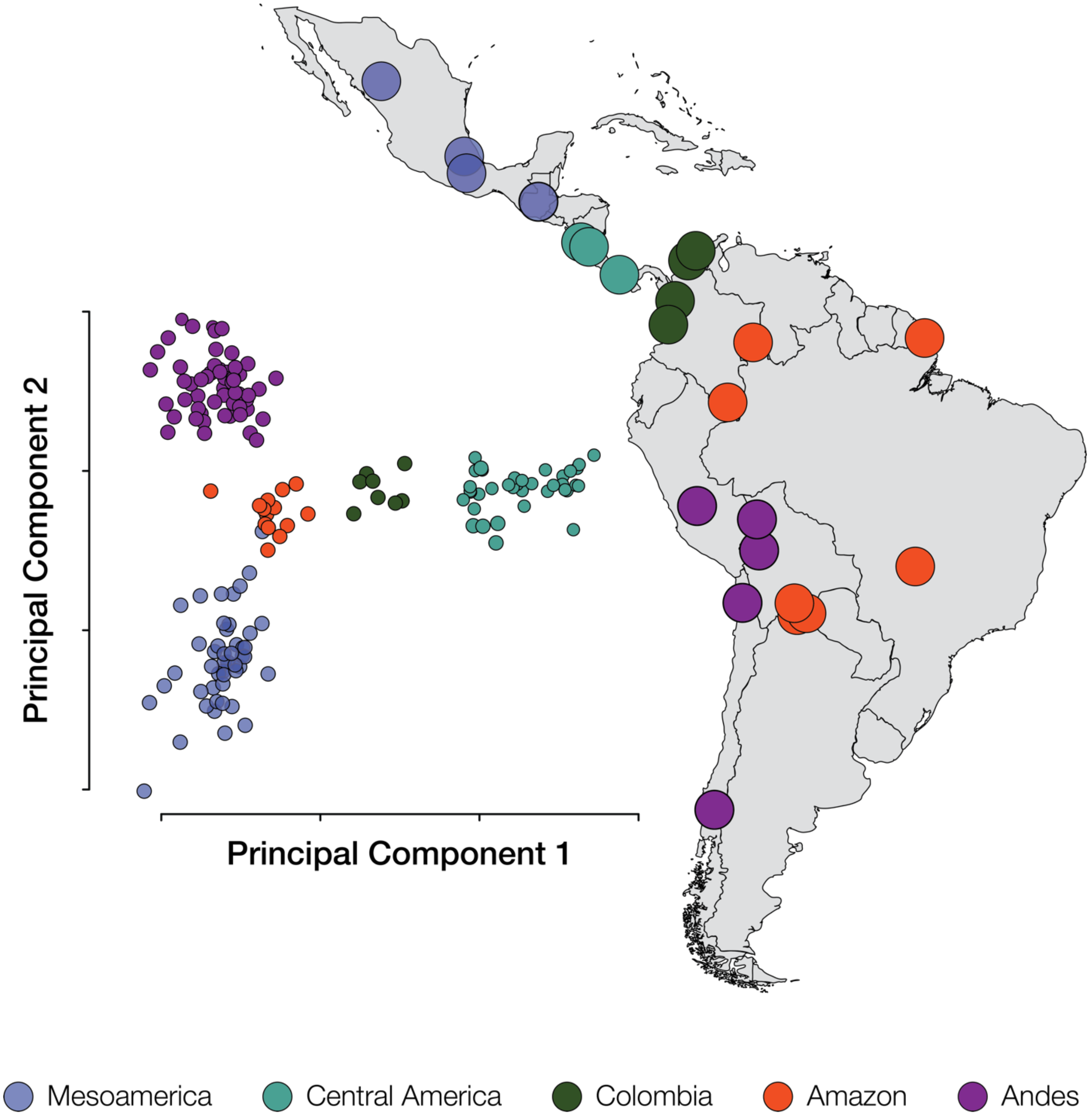

Figure S7. **Native American reference populations used for this study.** (A) The sampling locations of reference populations are shown, with respect to modern Latin American countries, along with the locations of the five main geographic/genetic groups: Mesoamerica (light blue), Central America (teal), Colombia (green), Amazon (orange), Andes (purple). (B) Principal components analysis (PCA) showing the genetic relationships among Native American reference population samples from the main groups.

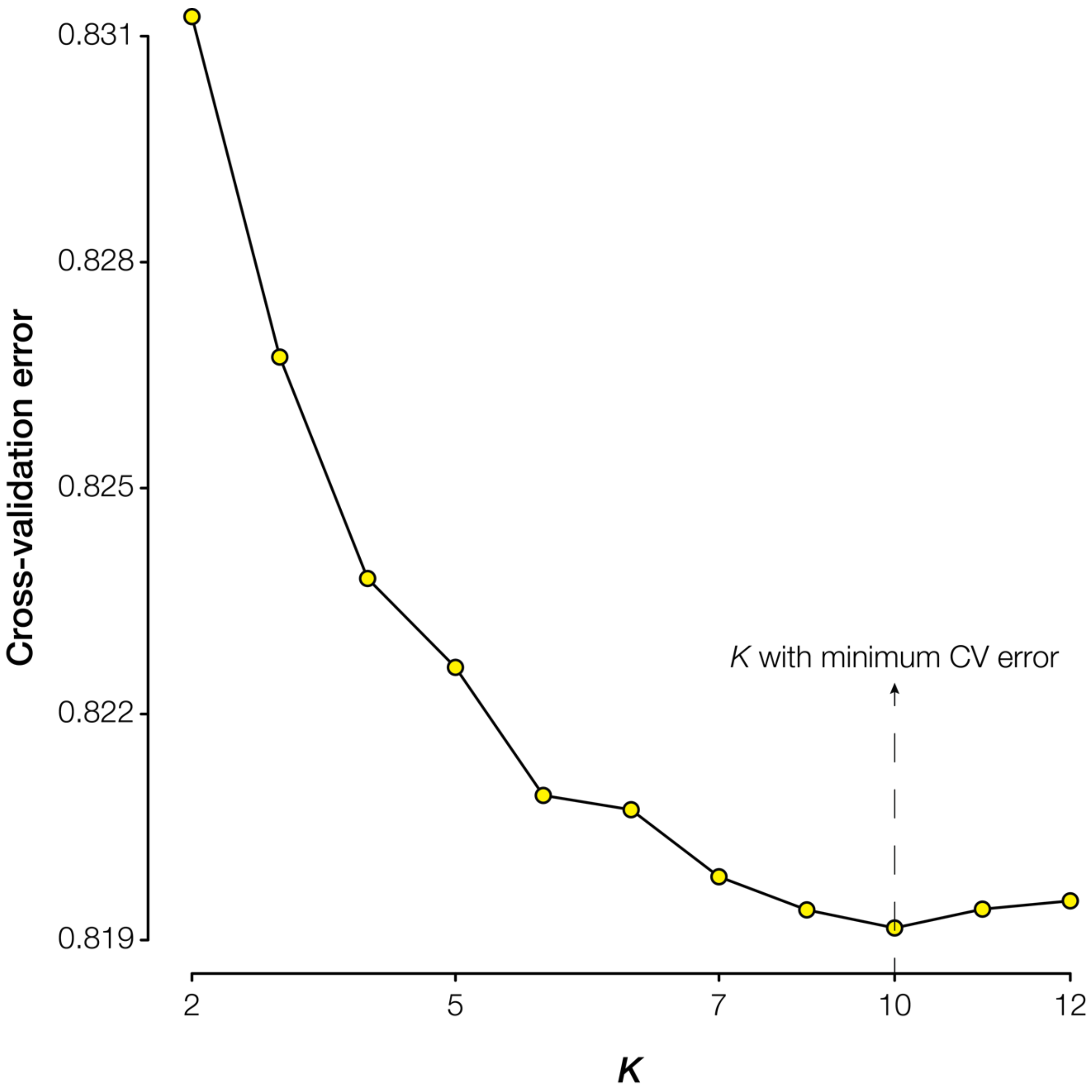

Figure S8. **Cross-validation error values (y-axis) for AMDIXTURE run over a range of ancestry component values K=2-12 on Native American ancestry.** The optimal value of K=10 was chosen for analysis Native American ancestry and admixture.

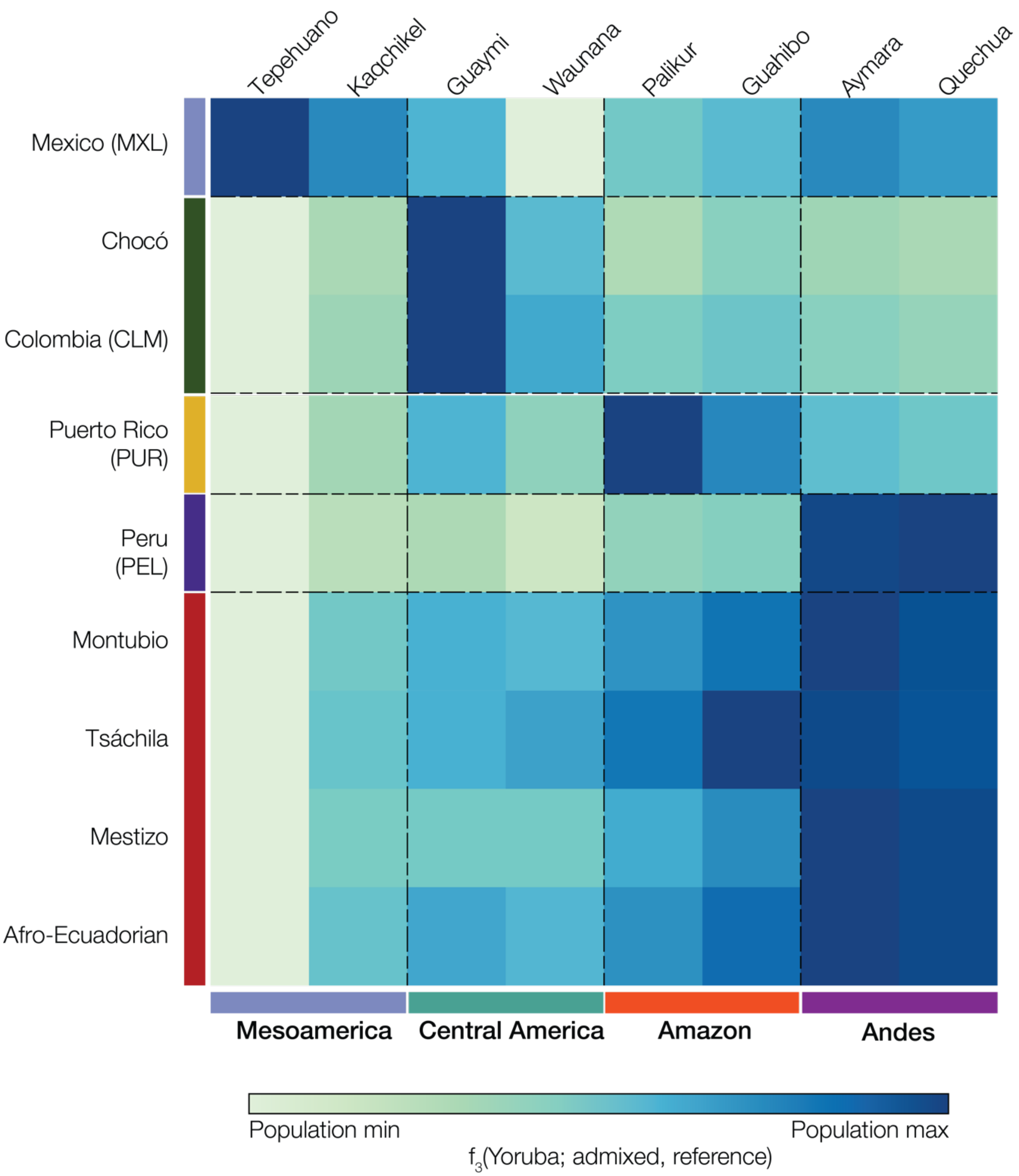

Figure S9. **Native American origins of Ecuadorian populations.** Phylogenetic similarity between the four Ecuadorian populations studied here, other admixed American populations, and Native American reference populations were assessed using the outgroup f3 statistic in the form shown. Similarity levels between admixed American populations and Native American reference populations are color coded as shown in the key.

Table S1. **Reference populations used in this study.** This table documents all the reference populations used for ancestry inference in this study – their continental and subcontinental population groups, sample size, and the technology used for genomic characterization.

#	Population	Continental group	Subcontinental group	Sample size	Genomic technology	Manuscript location	Reference
Whole genome sequence data (WGS) for continental and subcontinental ancestry inference							
1	Utah Residents (CEPH) with Northern and Western European Ancestry	European	North and Central Europe	99	WGS	Figure 1, Figure 4, Figure S2, Figure S3	1000 Genomes Project [1]
2	Finnish in Finland	European	North and Central Europe	99	WGS	Figure 4, Figure S3	1000 Genomes Project [1]
3	British in England and Scotland	European	North and Central Europe	91	WGS	Figure 1, Figure 4, Figure S2, Figure S3	1000 Genomes Project [1]
4	Toscani in Italia	European	South Europe	107	WGS	Figure 1, Figure 4, Figure S2, Figure S3	1000 Genomes Project [1]
5	Iberian Population in Spain	European	South Europe	107	WGS	Figure 1, Figure 4, Figure S2, Figure S3	1000 Genomes Project [1]
6	Gambian in Western Divisions in the Gambia	African	West Africa	113	WGS	Figure 4, Figure S3, Figure S5	1000 Genomes Project [1]
7	Mende in Sierra Leone	African	West Africa	85	WGS	Figure 1, Figure 4, Figure S2, Figure S3, Figure S5	1000 Genomes Project [1]

8	Yoruba in Ibadan, Nigeria	African	West Central Africa	108	WGS	Figure 1, Figure 4, Figure S2, Figure S3, Figure S5	1000 Genomes Project [1]
9	Esan in Nigeria	African	West Central Africa	99	WGS	Figure 1, Figure 4, Figure S2, Figure S3, Figure S5	1000 Genomes Project [1]
10	Luhya in Webuye, Kenya	African	East African	99	WGS	Figure 1, Figure 4, Figure S2, Figure S3, Figure S5	1000 Genomes Project [1]
11	Americans of African Ancestry in SW USA	Admixed American	Afro-descendant	61	WGS	Figure 1, Figure 4, Figure S2, Figure S3	1000 Genomes Project [1]
12	African Caribbeans in Barbados	Admixed American	Afro-descendant	96	WGS	Figure 1, Figure 4, Figure S2, Figure S3	1000 Genomes Project [1]
13	Puerto Ricans from Puerto Rico	Admixed American	Latin American	104	WGS	Figure 1, Figure 4, Figure 5, Figure S2, Figure S3	1000 Genomes Project [1]
14	Mexican Ancestry from Los Angeles USA	Admixed American	Latin American	64	WGS	Figure 1, Figure 4, Figure S2, Figure S3	1000 Genomes Project [1]
15	Colombians from Medellin, Colombia	Admixed American	Latin American	94	WGS	Figure 1, Figure 4, Figure 5, Figure S2, Figure S3	1000 Genomes Project [1]
16	Peruvians from Lima, Peru	Admixed American	Latin American	85	WGS	Figure 1, Figure 4, Figure 5,	1000 Genomes Project [1]

						Figure S2, Figure S3	
17	Han Chinese in Beijing, China	East Asian	Chinese	112	WGS	Figure 1, Figure S2, Figure S3	1000 Genomes Project [1]
18	Japanese in Tokyo, Japan	East Asian	Japanese	105	WGS	Figure 1, Figure S2, Figure S3	1000 Genomes Project [1]
**Whole genome genotype (WGG) arrays for subcontinental ancestry inference**							
**African subcontinental ancestry reference samples**							
19	Yacouba (Ivory Coast)	African	West Africa	20	Illumina HumanOmni Express-12 array	Figure 4, Figure S5	Patin et al., 2017 [2]
20	Ahizi (Ivory Coast)	African	West Africa	20	Illumina HumanOmni Express-12 array	Figure 4, Figure S5	Patin et al., 2017 [2]
21	Yoruba (Benin)	African	West Central Africa	20	Illumina HumanOmni Express-12 array	Figure 4, Figure S5	Patin et al., 2017 [2]
22	Bariba (Benin)	African	West Central Africa	20	Illumina HumanOmni Express-12 array	Figure 4, Figure S5	Patin et al., 2017 [2]
23	Fon (Benin)	African	West Central Africa	12	Illumina HumanOmni Express-12 array	Figure 4, Figure S5	Patin et al., 2017 [2]

24	Yaounde (Cameroon)	African	West Central Africa / Southwest Africa	39	Illumina HumanOmni Express-12 array	Figure 4, Figure S5	Patin et al., 2017 [2]
25	Kongo (Angola)	African	Southwest Africa	11	Illumina HumanOmni Express-12 array	Figure 4, Figure S5	Patin et al., 2017 [2]
26	Kimbundu (Angola)	African	Southwest Africa	18	Illumina HumanOmni Express-12 array	Figure 4, Figure S5	Patin et al., 2017 [2]
27	Ovimbundu (Angola)	African	Southwest Africa	16	Illumina HumanOmni Express-12 array	Figure 4, Figure S5	Patin et al., 2017 [2]
28	Umbundo (Angola)	African	Southwest Africa	5	Illumina HumanOmni Express-12 array	Figure 4, Figure S5	Patin et al., 2017 [2]
29	Bateke (Gabon)	African	Southwest Africa	54	Illumina HumanOmni Express-12 array	Figure 4, Figure S5	Patin et al., 2017 [2]
30	Nzebi (Gabon)	African	Southwest Africa	62	Illumina HumanOmni Express-12 array	Figure 4, Figure S5	Patin et al., 2017 [2]
31	Bapunu (Gabon)	African	Southwest Africa	53	Illumina HumanOmni	Figure 4, Figure S5	Patin et al., 2017 [2]

					Express-12 array		
32	Tsogo (Gabon)	African	Southwest Africa	65	Illumina HumanOmni Express-12 array	Figure 4, Figure S5	Patin et al., 2017 [2]
33	Eshira (Gabon)	African	Southwest Africa	41	Illumina HumanOmni Express-12 array	Figure 4, Figure S5	Patin et al., 2017 [2]
34	Galoa (Gabon)	African	Southwest Africa	50	Illumina HumanOmni Express-12 array	Figure 4, Figure S5	Patin et al., 2017 [2]
35	Orungu (Gabon)	African	Southwest Africa	22	Illumina HumanOmni Express-12 array	Figure 4, Figure S5	Patin et al., 2017 [2]
36	Duma (Gabon)	African	Southwest Africa	47	Illumina HumanOmni Express-12 array	Figure 4, Figure S5	Patin et al., 2017 [2]
37	Ndumu (Gabon)	African	Southwest Africa	38	Illumina HumanOmni Express-12 array	Figure 4, Figure S5	Patin et al., 2017 [2]
38	Obamba (Gabon)	African	Southwest Africa	46	Illumina HumanOmni Express-12 array	Figure 4, Figure S5	Patin et al., 2017 [2]

39	Benga (Gabon)	African	Southwest Africa	51	Illumina HumanOmni Express-12 array	Figure 4, Figure S5	Patin et al., 2017 [2]
40	Fang (Gabon)	African	Southwest Africa	69	Illumina HumanOmni Express-12 array	Figure 4, Figure S5	Patin et al., 2017 [2]
41	Badwee (Cameroon)	African	Southwest Africa	40	Illumina HumanOmni Express-12 array	Figure 4, Figure S5	Patin et al., 2017 [2]
42	Akele (Gabon)	African	Southwest Africa	49	Illumina HumanOmni Express-12 array	Figure 4, Figure S5	Patin et al., 2017 [2]
43	Okande (Gabon)	African	Southwest Africa	8	Illumina HumanOmni Express-12 array	Figure 4, Figure S5	Patin et al., 2017 [2]
44	Makina (Gabon)	African	Southwest Africa	45	Illumina HumanOmni Express-12 array	Figure 4, Figure S5	Patin et al., 2017 [2]
45	Bakota (Gabon)	African	Southwest Africa	56	Illumina HumanOmni Express-12 array	Figure 4, Figure S5	Patin et al., 2017 [2]
46	Bekwil (Gabon)	African	Southwest Africa	5	Illumina HumanOmni	Figure S5	Patin et al., 2017 [2]

					Express-12 array		
47	Eviya (Gabon)	African	Southwest Africa	31	Illumina HumanOmni Express-12 array	Figure S5	Patin et al., 2017 [2]
48	Shake (Gabon)	African	Southwest Africa	52	Illumina HumanOmni Express-12 array	Figure 4, Figure S5	Patin et al., 2017 [2]
49	Babongo (east) (Gabon)	African	Southwest Africa / Rain Forest Hunter Gatherer	40	Illumina HumanOmni Express-12 array	Figure 4, Figure S5	Patin et al., 2017 [2]
50	Biaka (Central African Republic)	African	Rain Forest Hunter Gatherer	20	Illumina HumanOmni Express-12 array	Figure 4, Figure S5	Patin et al., 2017 [2]
51	Baka (Cameroon)	African	Rain Forest Hunter Gatherer	117	Illumina HumanOmni Express-12 array	Figure S5	Patin et al., 2017 [2]
52	Bakoya (Gabon)	African	Rain Forest Hunter Gatherer	25	Illumina HumanOmni Express-12 array	Figure S5	Patin et al., 2017 [2]
53	Bezan (Cameroon)	African	Rain Forest Hunter Gatherer	26	Illumina HumanOmni Express-12 array	Figure S5	Patin et al., 2017 [2]

54	Mbuti (Democratic Republic of Congo)	African	Rain Forest Hunter Gatherer	13	Illumina HumanOmni Express-12 array	Figure S5	Patin et al., 2017 [2]
55	Batwa (Democratic Republic of Congo)	African	Rain Forest Hunter Gatherer	2	Illumina HumanOmni Express-12 array	Figure S5	Patin et al., 2017 [2]
**Native American subcontinental ancestry reference samples**							
56	Tepehuano (Mexico)	Native American	Mesoamerica	25	Illumina HumanHap550 V3.0 array	Figure 5, Figure S7, Figure S9	Reich et al., 2012 [3]
57	Mixe (Mexico)	Native American	Mesoamerica	17	Illumina 610-Quad array	Figure 5, Figure S7	Reich et al., 2012 [3]
58	Mixtec (Mexico)	Native American	Mesoamerica	5	Illumina 610-Quad array	Figure 5, Figure S7	Reich et al., 2012 [3]
59	Kaqchikel (Guatemala)	Native American	Mesoamerica	13	Illumina 610-Quad array	Figure 5, Figure S7, Figure S9	Reich et al., 2012 [3]
60	Guaymi (Costa Rica)	Native American	Central America	5	Illumina 610-Quad array	Figure 5, Figure S7, Figure S9	Reich et al., 2012 [3]
61	Teribe (Costa Rica)	Native American	Central America	3	Illumina 610-Quad array	Figure 5, Figure S7	Reich et al., 2012 [3]
62	Cabecar (Costa Rica)	Native American	Central America	31	Illumina 610-Quad array	Figure 5, Figure S7	Reich et al., 2012 [3]
63	Embera (Colombia)	Native American	Colombia	5	Illumina 610-Quad array	Figure 5, Figure S7	Reich et al., 2012 [3]
64	Kogi (Colombia)	Native American	Colombia	4	Illumina 610-Quad array	Figure S7	Reich et al., 2012 [3]
65	Chocó (Colombia)	Admixed American	Colombia	94	Illumina HumanOmni Express-24	Figure 5, Figure S7	Conley et al., 2017[4]

66	Waunana (Colombia)	Native American	Colombia	3	Illumina 610-Quad array	Figure S7, Figure S9	Reich et al., 2012 [3]
67	Wayuu (Colombia)	Native American	Colombia	11	Illumina 610-Quad array	Figure S7	Reich et al., 2012 [3]
68	Piapoco (Colombia)	Native American	Amazon	7	Illumina 610-Quad array	Figure 5, Figure S7	Reich et al., 2012 [3]
69	Guahibo (Colombia)	Native American	Amazon	6	Illumina 610-Quad array	Figure 5, Figure S7, Figure S9	Reich et al., 2012 [3]
70	Guarani (Paraguay & Argentina)	Native American	Amazon	6	Illumina 610-Quad array	Figure 5, Figure S7	Reich et al., 2012 [3]
71	Palikur (Guiana)	Native American	Amazon	3	Illumina 610-Quad array	Figure 5, Figure S7, Figure S9	Reich et al., 2012 [3]
72	Ticuna (Colombia)	Native American	Amazon	6	Illumina 610-Quad array	Figure S7	Reich et al., 2012 [3]
73	Toba (Argentina)	Native American	Amazon	4	Illumina 610-Quad array	Figure S7	Reich et al., 2012 [3]
74	Wichi (Argentina)	Native American	Amazon	5	Illumina 610-Quad array	Figure S7	Reich et al., 2012 [3]
75	Aymara (Bolivia & Chile)	Native American	Andes	23	Illumina 610-Quad array	Figure 5, Figure S7, Figure S9	Reich et al., 2012 [3]
76	Inga (Colombia)	Native American	Andes	9	Illumina 610-Quad array	Figure S7	Reich et al., 2012 [3]
77	Quechua (Bolivia & Peru)	Native American	Andes	40	Illumina 610-Quad array	Figure 5, Figure S7, Figure S9	Reich et al., 2012 [3]
78	Hulliche (Chile)	Native American	Andes	4	Illumina 610-Quad array	Figure S7	Reich et al., 2012 [3]

WGS – Whole Genome Sequencing

References:

1. Genomes Project C, Auton A, Brooks LD, et al. A global reference for human genetic variation. *Nature* 2015; **526**(7571): 68-74.
2. Patin E, Lopez M, Grollemund R, et al. Dispersals and genetic adaptation of Bantu-speaking populations in Africa and North America. *Science* 2017; **356**(6337): 543-6.
3. Reich D, Patterson N, Campbell D, et al. Reconstructing Native American population history. *Nature* 2012; **488**(7411): 370-4.
4. Conley AB, Rishishwar L, Norris ET, et al. A Comparative Analysis of Genetic Ancestry and Admixture in the Colombian Populations of Choco and Medellin. *G3 (Bethesda)* 2017; **7**(10): 3435-47.
